# Urban effluents override drying legacy in stream biofilm bacterial communities

**DOI:** 10.1093/ismeco/ycag270

**Published:** 2026-09-23

**Authors:** Javier Ortiz-Rivero, Paula Siñeriz, Sergi Sabater, Anna Freixa

**Affiliations:** Catalan Institute for Water Research (ICRA-CERCA), Parc Científic i Tecnològic de la Universitat de Girona, Carrer Emili Grahit 101, Girona 17003, Spain; University of Girona, Plaça de Sant Domènec 3, Girona 17004, Spain; Catalan Institute for Water Research (ICRA-CERCA), Parc Científic i Tecnològic de la Universitat de Girona, Carrer Emili Grahit 101, Girona 17003, Spain; Catalan Institute for Water Research (ICRA-CERCA), Parc Científic i Tecnològic de la Universitat de Girona, Carrer Emili Grahit 101, Girona 17003, Spain; Institute of Aquatic Ecology, University of Girona, Campus de Montilivi, Girona 17071, Spain; Catalan Institute for Water Research (ICRA-CERCA), Parc Científic i Tecnològic de la Universitat de Girona, Carrer Emili Grahit 101, Girona 17003, Spain

**Keywords:** bacterial diversity, climate change, community resilience, environmental history, fluvial biofilm, urban pollution

## Abstract

Bacterial communities in stream biofilms play a fundamental role in the ecological functioning of fluvial ecosystems, contributing to nutrient cycling, organic matter decomposition, and primary production, while also serving as indicators of ecosystem health. In Mediterranean streams, these communities are increasingly exposed to multiple sequential stressors of varying intensity, such as drying and chemical pollution, yet their combined effects remain poorly understood. Here, we conducted a field experiment in four streams by colonizing artificial substrates, which were subsequently subjected to contrasting drying regimes (intermittent vs. continuous) and later translocated to reaches receiving urban effluents. Bacterial communities were characterized using high-throughput sequencing of the 16S rRNA gene. Both stressors significantly modified community diversity and composition, with consistent responses across streams. Continuous drying acted as a strong environmental filter, reducing diversity and favoring stress-tolerant taxa such as Bacilli and Actinobacteria while decreasing the relative abundance of Cyanobacteria. In contrast, effluent exposure promoted community homogenization and recolonization by effluent-derived taxa, largely dominated by Cyanobacteria and Gammaproteobacteria. Across both intermittent and continuous regimes, sequential exposure to drying followed by urban effluents further shaped bacterial communities. However, legacy effects of drying were generally weak and largely overridden by effluent inputs. Although some early-colonizing, stress-tolerant taxa persisted, community reorganization was mainly driven by effluent-derived colonization and local environmental conditions. Overall, these results show that the sequence and intensity of disturbances, together with initial community composition, regulate microbial resilience and community reorganization, with clear implications for the functioning of river ecosystems.

## Introduction

Bacterial biofilm communities are pivotal to fundamental biogeochemical processes in river ecosystems and display quick responses to environmental changes [1, 2]. Their high functional and genetic diversity [3] supports key activities such as nutrient cycling, primary production, or organic matter decomposition [4–6]. Human-derived stressors, such as pesticides [7], pharmaceuticals [8], microplastics [9], and intense droughts or floods [10, 11] strongly influence bacterial diversity and community composition, as well as their functions in the ecosystem processes [12, 13].

In particular, the increasing frequency and intensity of dry periods driven by climate change [11] are frequently associated with the accumulation of urban pollutants [14], largely due to reduced dilution capacity under low-flow conditions [15]. In river ecosystems, intense dry periods may lead to streambed drying and impose severe physiological stress or even cell death on bacterial biofilm communities [16, 17]. Nevertheless, certain taxa can persist under these conditions, exhibiting high resistance and functional plasticity to drying [18, 19] which enables biofilms to maintain essential ecological functions under such conditions [20, 21]. Dry periods often facilitate the transient incorporation of terrestrial taxa, such as Actinobacteria, spore-forming Firmicutes, or specific members of Alphaproteobacteria [18, 19]. As a result, these taxa may dominate fluvial biofilms during the dry periods and subsequently contribute to temporal shifts in bacterial community composition upon rewetting.

On the other hand, urban effluents entering streams via wastewater treatment plants (WWTPs) can strongly influence bacterial composition and diversity, primarily by promoting the arrival of opportunistic taxa adapted to the presence of micropollutants or nutrient-enriched conditions in the water [22–24]. This adaptative response often leads to changes in benthic biofilm, particularly with representative bacteria such as *Mycobacterium*, *Nitrosomonas*, and *Gordonia*, whose contribution reflects a shift toward pollutant-tolerant and metabolically versatile communities [23]. Their success is largely driven by the composition and concentration of discharged compounds, the dilution capacity, and the local environmental conditions of the receiving river [25, 26]. Additionally, WWTP discharges supply allochthonous microorganisms that may further enhance community tolerance to chemical stressors [27, 28]. Overall, WWTP effluents exert a strong control over the structure of bacterial biofilm communities in receiving river ecosystems [22].

These stressors rarely occur in isolation; rather often occur simultaneously or sequentially, advancing the complexity of the ecological responses [29]. Previous studies have already reported either the effects of streambed drying [18, 30] or the impacts of WWTP effluents [28, 31] on stream bacterial communities, their joint or sequential effects have not been considered beyond controlled laboratory conditions [19, 32]. However, laboratory approaches usually focus on a single biofilm community and therefore do not capture the intrinsic variability existing among natural communities. In this study, we address this limitation by evaluating the responses of several stream biofilms submitted to a sequence of stressors. To this end, we conducted a field experiment using biofilms from four Mediterranean streams, differing in nutrient concentrations, and geomorphological characteristics. Biofilm communities were first exposed to contrasting drying regimes (intermittent vs continuous) and subsequently translocated to downstream reaches receiving urban effluents. This experimental design enabled us to evaluate whether drying influenced biofilm responses to urban pollution, and whether such effects are consistent across systems [33, 34]. Accordingly, we expected bacterial communities to shift in diversity and composition under individual and sequential stressors, relative to unimpacted biofilms.

Based on this, we hypothesized that: (i) Streambed drying would reduce bacterial richness, diversity, and evenness and shift community composition, [35, 36], with stronger effects under continuous rather than intermittent drying (INT-DRY). (ii) Exposure to WWTP effluents would increase bacterial richness, diversity, and evenness through recolonization processes, driven by abrupt changes in community structure [19, 37]. (iii) Bacterial communities previously exposed to drying would respond differently to WWTP effluents than communities without prior drying exposure, potentially reflecting legacy effects of drying [34, 35]. (iv) Depending on stressor intensity, the bacterial compositional changes would differ in the relative contribution of nestedness and turnover components of beta diversity [38, 39]. (v) Finally, the responses of biofilm bacterial communities to drying and urban effluents would vary among streams according to their initial community composition, structure, and local environmental conditions [40–42].

Overall, this study aims to characterize how bacterial biofilm communities respond to the sequential exposure to two stressors: streambed drying and the impact of urban effluents. By examining natural communities, we provide a realistic view of how global change and anthropogenic pressures interact to shape microbial assemblages in freshwater ecosystems and their potential ecosystem-level consequences.

## Materials and methods

### Field site and experimental design

Experiments were conducted in four second- to third-order streams (Strahler classification) in NE Catalonia, Spain, with a typical Mediterranean regime during spring and early summer (2022: Arbúcies and Llémena; 2023: Major and Terri) (Supplementary Fig. S1). Two reaches per stream were selected: an upstream unimpacted reach and a downstream reach receiving treated urban effluent (WWTP), separated by distances ranging from 0.55 to 7.8 km.

Artificial substrata consisting of bricks with 25 ceramic tiles (1 cm^2^) were deployed in the unimpacted reaches and submerged for 4 weeks to allow biofilm colonization, a period sufficient to obtain biofilms comparable to natural assemblages [43] (‘Unimpacted-T1’; Table 1). Two subsets of biofilm-covered bricks were then exposed on the riverbank to simulate drying stress at two intensity levels [44] for 3 weeks: INT-DRY(three cycles of 5 days air exposure followed by 2 days rehydration) and continuous drying (‘CON-DRY’). A control subset remained submerged (‘Unimpacted-T2’). Bricks were collected at the end of the drying period for bacterial analysis.

**Table 1:** Experimental treatments and corresponding biofilm targets at three sampling times: first sampling (T1: (after 4 weeks of colonization), second sampling (T2: after 3 weeks of exposure to drying), and third sampling (T3: after 3 weeks of exposure to urban effluents).

Treatment block	Treatment	Target
Unimpacted	UN (T1)	Biofilm after the colonization period at the unimpacted reach(first sampling)
	UN (T2)	Biofilm remaining in the stream at the unimpacted reach(second sampling)
	UN (T3)	Biofilm remaining in the stream at the unimpacted reach(third sampling)
Drying	INT-DRY (T2)	Biofilm subjected to INT-DRY
	CON-DRY (T2)	Biofilm subjected to CON-DRY
Impacted	IMP (T3)	Biofilm translocated to the effluent-impacted reach (previously located at the unimpacted reach)
	IMP INT-DRY (T3)	Biofilm translocated to the effluent-impacted reach after INT-DRY
	IMP CON-DRY (T3)	Biofilm translocated to the effluent-impacted reach after CON-DRY

Finally, bricks from INT-DRY, CON-DRY, and Unimpacted-T2 were translocated to downstream impacted reaches for 3 weeks to assess the effects of wastewater effluent. At the end of this period (T3), biofilm samples were collected from the unimpacted reach (‘Unimpacted-T3’) and from the translocated bricks in the impacted reach (‘IMP’, ‘IMP INT-DRY’, ‘IMP CON-DRY’; Table 1).

### Physicochemical water characterization

Water physicochemical variables were measured in the unimpacted reach at all sampling times (T1, T2, and T3) and in the impacted reach at T3. Temperature, pH, conductivity, and dissolved oxygen were measured *in situ* using portable probes (WTW, Weilheim, Germany). Water samples for chemical analyses were collected to determine nutrients and dissolved organic carbon (DOC) concentrations, and were filtered *in situ* using a sterile disposable syringe and a single-use filter for each sample to avoid cross-contamination. Samples for nutrient analysis were filtered through 0.22 μm nylon filters (Whatman, Kent, UK) and subsequently analyzed for anions (NO₂^−^, NO₃^−^, PO₄^3−^, Cl^−^, SO₄^2−^) and cations (NH₄^+^, Na^+^, K^+^, Mg^2+^, Ca^2+^) using a Dionex ICS-5000 ion chromatograph (Dionex, Sunnyvale, USA). Water samples for DOC analysis were filtered through 0.7 μm precombusted glass fiber filters (Whatman GF/C, Kent, UK), and DOC concentrations were determined using a Shimadzu TOC-V CSH analyzer coupled to a TNM-1 (Total Nitrogen Module) (Shimadzu Corporation, Kyoto, Japan).

### DNA extraction and high-throughput sequencing

Biofilm samples were collected by scraping the biofilm from four randomly selected ceramic tiles using a sterile brush. The scraped biofilm was resuspended in filtered stream water and processed in triplicate for each treatment. Genomic DNA was extracted using the FastDNA® Spin Kit for Soils (MP Biomedicals) following the manufacturer’s instructions with minor modifications. Briefly, ~2 ml of biofilm was centrifuged (10 000 rpm, 30 s) to obtain a pellet for extraction. DNA was eluted in 50 μl of RNase-free water. Concentration and purity were assessed using a Qubit 2.0 fluorometer (Invitrogen) and a NanoDrop 2000 spectrophotometer (Thermo Fisher Scientific), respectively.

The V4 region of the 16S rRNA gene was amplified following the Earth Microbiome Project protocol using primers 515F/806R [45]. Polymerase chain reaction (PCR) conditions consisted of an initial denaturation at 94°C for 3 min, followed by 35 cycles (94°C for 45 s, 50°C for 60 s, 72°C for 90 s) and a final extension at 72°C for 10 min. Amplification was verified by agarose gel electrophoresis. Amplicon libraries were prepared at the SGIker Genomic Facility (University of the Basque Country, Spain) and sequenced on an Illumina MiSeq platform (2 × 250 bp).

### 16S rRNA data processing

Sequencing data were processed using the Divisive Amplicon Denoising Algorithm [46], which identifies Amplicon Sequence Variants (hereafter referred to as ASVs) differing by a single nucleotide. This allows the identification of a greater number of genetic variants compared to the inference of Operational Taxonomic Units or other similar methods [47]. Demultiplexing, data quality control, data filtering, clustering into ASVs, and construction of abundance tables containing the inferred bacterial ASVs and their corresponding read counts per sample were performed in QIIME2 [48]. To reduce potential sequencing artifacts and remove rare ASVs prior to downstream analyses, the feature table was converted into Biological Observation Matrix (BIOM) format and filtered by retaining only ASVs with at least 100 total reads, present in a minimum of four samples, and classified at least at the phylum level. Sequences assigned to Eukarya and Archaea were excluded from further analyses. Taxonomic classification of the bacterial ASVs was performed using the SILVA reference database (version 138, available at https://www.arb-silva.de/) [49]. The resulting ASV, taxonomy, and metadata tables were exported for subsequent statistical analyses in R 4.6.1 [50].

### Microbial diversity and composition analyses

Samples were grouped into eight experimental treatments within each stream and three treatment blocks (Table 1): unimpacted (UN-T1, UN-T2, UN-T3), drying (INT-DRY, CON-DRY), and impacted (IMP, IMP-INT-DRY, IMP-CON-DRY). A total of 96 libraries were generated, and one library containing only 484 reads was excluded from downstream analyses because of its exceptionally low sequencing depth (Supplementary Fig. S2). The remaining 95 libraries yielded an average sequencing depth of 50 996 reads per sample. Alpha diversity (ASV richness, Shannon index, Pielou’s evenness) was calculated using the ASV table rarefied to a depth of 8601 reads per sample to ensure comparability across samples. Treatment effects on these alpha diversity metrics were evaluated using analysis of variance (ANOVA) or Kruskal–Wallis tests, followed by Tukey’s Honestly Significant Difference (HSD) or Dunn’s post hoc pairwise comparisons, respectively. Community composition was summarized at the class level, with dominant taxa identified at the family level using Similitary Percentage (SIMPER) analysis. Differences among treatments were assessed using Bray–Curtis dissimilarities and visualized with non-metric multidimensional scaling (NMDS). Statistical differences in community composition were evaluated using permutational multivariate analysis of variance (PERMANOVA) and analysis of similarities (ANOSIM), followed by post hoc pairwise comparisons where appropriate. Beta diversity was partitioned into turnover and nestedness components, and environmental influences were evaluated with distance-based redundacy analysis (dbRDA). Shared and unique ASVs were visualized with Venn and UpSet plots. All statistical analyses and figures were performed in R (version 4.6.1). Detailed information on the analytical workflow, packages, and visualization procedures is provided in the Supplementary Material (Appendix S1).

## Results

### Background environmental gradients shaping biofilm communities

Baseline physicochemical conditions at the unimpacted sites varied among the four streams (Supplementary Table S1). Terri exhibited the highest conductivity (1393–1512 μS cm^−1^) and ionic concentrations (Cl^−^, SO₄^2−^, Na^+^, Mg^2+^, Ca^2+^), whereas Arbúcies and Major were characterized by lower conductivity values (226–248 μS cm^−1^). Water temperature was comparatively higher in Terri and Arbúcies and lower in Major, while dissolved oxygen displayed the opposite pattern. DOC concentrations remained low (0.88–2.23 mg C l^−1^) and showed little variation across the sampling times. Downstream of the WWTP, physicochemical measurements generally showed higher conductivity, ionic concentrations, DOC, and nutrient levels, together with lower pH and dissolved oxygen than at the corresponding unimpacted sites, although the magnitude of these differences varied among streams (Supplementary Table S1).

dbRDA analysis showed that the biofilm bacterial community structure at unimpacted sites was strongly associated with physicochemical variables (Supplementary Fig. S3, Supplementary Table S2). The first two dbRDA axes explained ~72% of the variation (dbRDA1: 37.25%; dbRDA2: 34.68%), reflecting mainly spatial differences among streams. dbRDA1 separated Llémena communities, associated with higher temperature and cation concentrations, from Major and Arbúcies communities, linked to higher dissolved oxygen and DOC. dbRDA2 distinguished the Terri stream, associated with higher K^+^ and pH.

### Bacterial community composition and relative abundance

The unimpacted biofilm communities (UN-T1, UN-T2, UN-T3) were mainly composed of Alphaproteobacteria and Cyanobacteriia, although their relative abundances differed among streams. In the Terri stream, Cyanobacteriia represented the largest fraction of the community (37%), followed by Alphaproteobacteria (25%). In contrast, Alphaproteobacteria represented the largest fraction in the Llémena stream (30%), followed by Cyanobacteriia (23%), whereas Verrucomicrobiae accounted for ~6% of the community. In the Arbúcies and Major streams, Alphaproteobacteria, Cyanobacteriia, and Gammaproteobacteria showed comparable relative abundances (Fig. 1).

**Figure 1:**
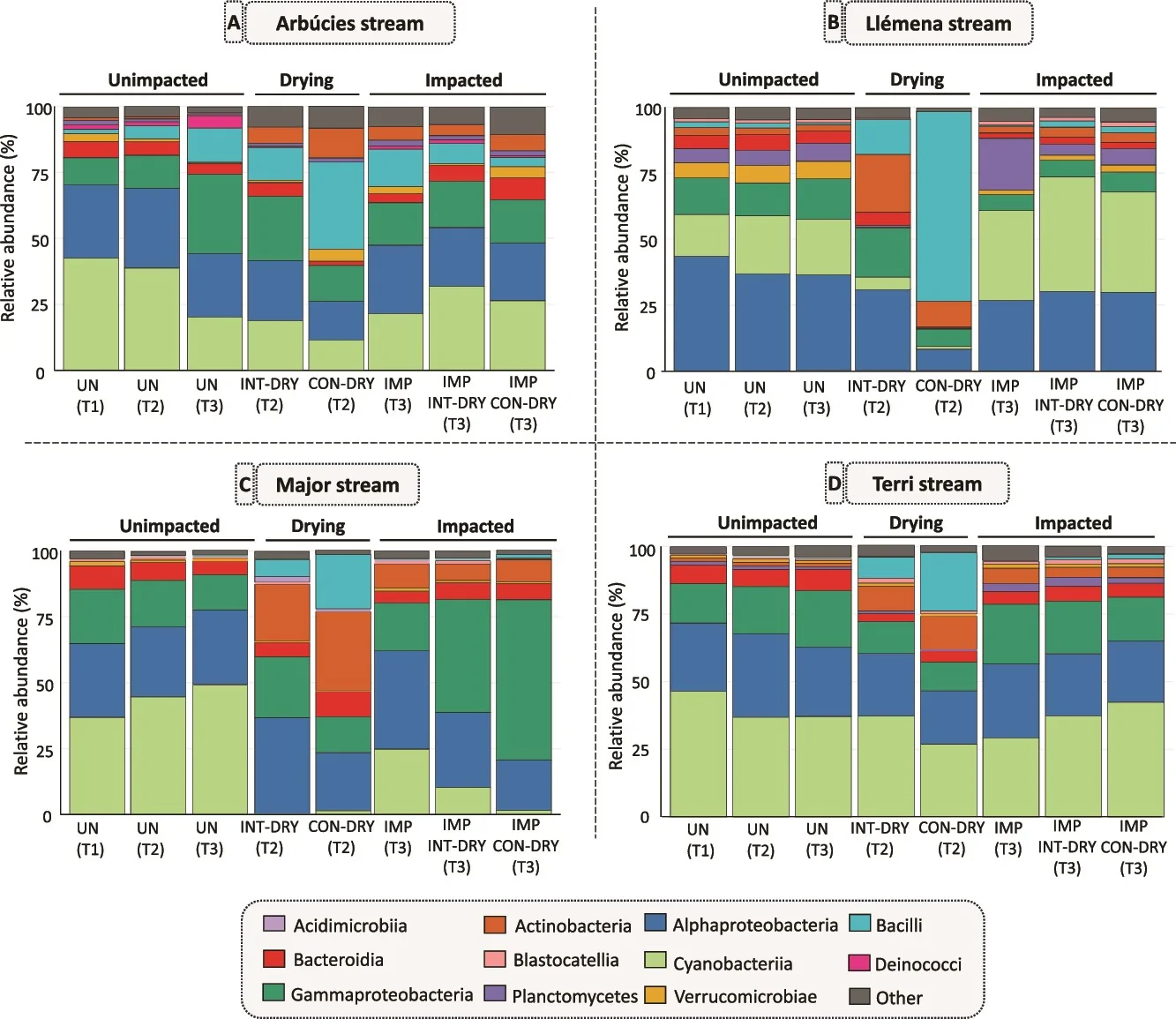
Bacterial composition of biofilm communities from the four streams: (A) Arbúcies, (B) Llémena, (C) major, and (D) Terri across experimental treatments; stacked bars show the relative abundances (%) of bacterial classes under unimpacted (UN T1, UN T2, UN T3), drying (INT-DRY, CON-DRY), and impacted treatments (IMP, IMP INT-DRY, IMP CON-DRY); for each stream, the 10 most abundant bacterial classes are displayed, while less abundant taxa are grouped under “other.”

Drying (CONT-DRY and INT-DRY) imposed marked increases in Bacilli and Actinobacteria across all bacterial communities, although their relative importance differed among stream biofilms, with statistically significant increases in Actinobacteria in all streams and in Bacilli in Llémena, Major, and Terri (Fig. 1, Supplementary Table S3). In the Llémena stream, Bacilli and Actinobacteria reached 72% and 22% of the community, respectively. In the Arbúcies and Terri streams, Bacilli increased more moderately, reaching 33% and 15%, respectively. Actinobacteria reached up to 26% in the Major stream and remained at lower relative abundances in the Arbúcies and Terri streams. Drying also induced stream-specific shifts in other dominant bacterial classes, including significant declines in Alphaproteobacteria in the Llémena and Terri streams, Cyanobacteriia in the Major and Llémena streams, and Verrucomicrobiae in the Llémena stream (Fig. 1, Supplementary Table S3).

Urban effluents (IMP, IMP-INT-DRY, IMP CON-DRY) further modified the taxonomic composition of biofilm communities, although the response differed markedly among streams. In the Arbúcies stream, community composition remained largely unchanged, with Actinobacteria persisting after wastewater exposure. In the Llémena stream, Cyanobacteriia represented the largest fraction of the community (38%), accompanied by a significant decline in Bacilli, whereas Planctomycetes accounted for ~10% of the community. In the Major stream, Gammaproteobacteria dominated the impacted communities, reaching up to 61% relative abundance, whereas Bacilli significantly declined following wastewater exposure (Fig. 1, Supplementary Table S3). In the Terri stream, the taxonomic composition of impacted biofilms closely resembled that of the unimpacted biofilms, despite significant changes in the relative abundance of several bacterial classes (Supplementary Table S3).

Venn diagrams showed a substantial overlap of ASVs between unimpacted, drying, and impacted biofilms, consistently across all streams (Fig. 2, Supplementary Fig. S4, Supplementary Table S4). At the local scale (Fig. 2), the number of shared ASVs was highest in the Terri (570; 48%) and Arbúcies (498; 46%), and lowest in the Llémena (277; 30%) and Major (222; 26%). The impacted reaches consistently presented the highest proportion of exclusive ASVs (43%–56%), with the maximum in Arbúcies (56%). In contrast, the lowest number of exclusive ASVs (11%–17%) was under drying treatments (except in the Terri, 30%). Pairwise overlaps were larger between unimpacted and impacted (32%–53%) than between the unimpacted and drying (5%–13%), and intermediate between the drying–impacted (9%–16%). At the regional scale (Supplementary Fig. S4), patterns were largely coincident with local trends, with effluent-impacted reaches harboring the highest number of exclusive ASVs and drying biofilms consistently showing the lowest values.

**Figure 2:**
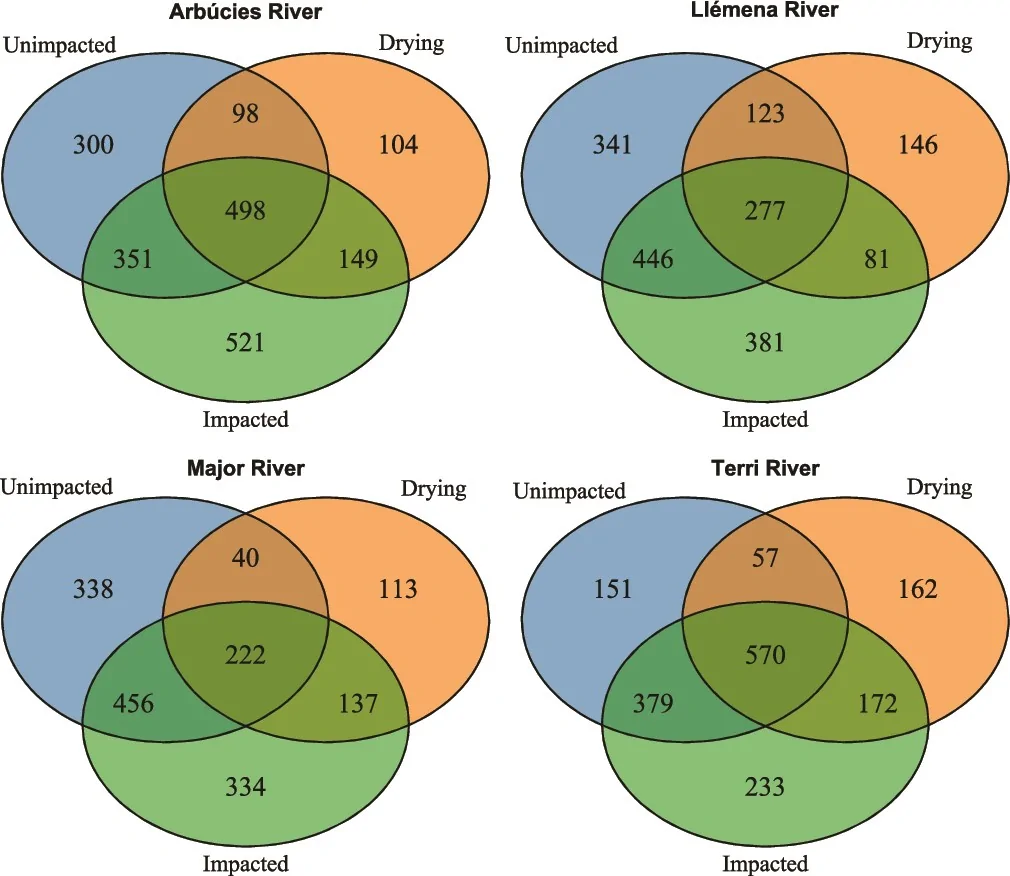
Venn diagrams showing the number of shared and exclusive bacterial taxa (ASVs) in biofilm communities from the four streams (Arbúcies, Llémena, Major, and Terri) under unimpacted, drying, and impacted conditions; numbers indicate taxa exclusive to each treatment block, as well as those shared among groups within each stream.

### Alpha and Beta diversities analysis

#### Alpha diversity patterns

At the unimpacted sites, alpha diversity showed stream-specific patterns (Fig. 3, Supplementary Table S4). Terri biofilms had the highest observed richness (439–470 ASVs) and diversity values, whereas Major biofilms had the lowest richness (246–373 ASVs) and Shannon diversity (4.28–4.85). Llémena biofilms were characterized by relatively high evenness (0.84–0.87) and diversity (4.92–5.14) despite their moderate richness.

**Figure 3:**
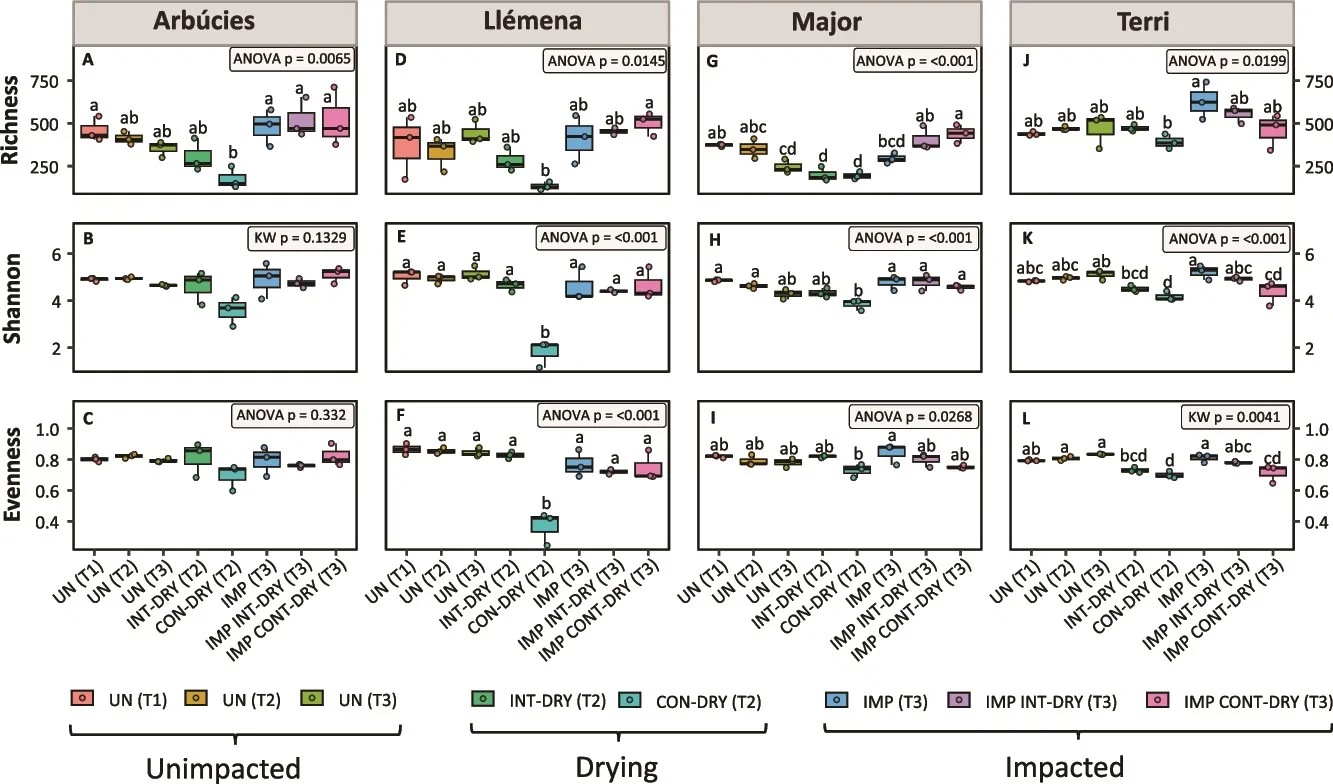
Alpha diversity metrics of biofilm bacterial communities for each stream across experimental treatments; panels show richness, Shannon diversity, and Pielou’s evenness under unimpacted (UN T1, UN T2, UN T3), drying (INT-DRY T2, CON-DRY T2), and impacted treatments (IMP T3, IMP INT-DRY T3, IMP CON-DRY T3); different letters indicate significant differences among treatments within each stream (*P* < .05), based on ANOVA or Kruskal–Wallis tests.

Within each stream, drying generally reduced alpha diversity, with the strongest effects observed under CONT-DRY. This decline was particularly pronounced in the Llémena (richness: 133 ASVs; Shannon: 1.80; evenness: 0.37), while CON-DRY also markedly reduced diversity indices in the Arbúcies and Major (richness: 176 and 194 ASVs; Shannon: 3.58 and 3.84, respectively). In contrast, Terri maintained comparatively high richness and diversity under both intermittent and CON-DRY (richness: 392–472 ASVs; Shannon: 4.18–4.50) (Fig. 3, Supplementary Table S5).

Following exposure to urban effluents, richness generally increased within each stream, reaching a maximum of 630 ASVs in Terri. In Arbúcies, Llémena, and Major, the highest richness values were observed in biofilms subjected to CON-DRY followed by urban effluent exposure (IMP CONT-DRY). In Arbúcies and Major, diversity and evenness remained similar to those observed in the corresponding unimpacted biofilms. In Llémena, diversity and evenness partially recovered after drying (Shannon: 4.40–4.65; evenness: 0.72–0.77), although these values remained lower than those observed under unimpacted conditions (Fig. 3, Supplementary Table S5).

#### Beta diversity patterns

##### Local beta diversity patterns

Bray–Curtis dissimilarities revealed significant compositional shifts among unimpacted, drying, and impacted biofilms, with unimpacted biofilms forming distinct clusters at the local scale (Fig. 4). These patterns were statistically supported by ANOSIM (Supplementary Table S6), with the strongest separation observed in Llémena (*R* = 0.937), followed by Major (*R* = 0.888) and Terri (*R* = 0.880), and lower in Arbúcies (*R* = 0.665). PERMANOVA indicated that treatment blocks accounted for a substantial fraction of compositional variation across streams (*R*^2^ = 0.322–0.550; Supplementary Table S5), with post hoc pairwise tests confirming significant differences among blocks (*P* < .001; Supplementary Table S7).

**Figure 4:**
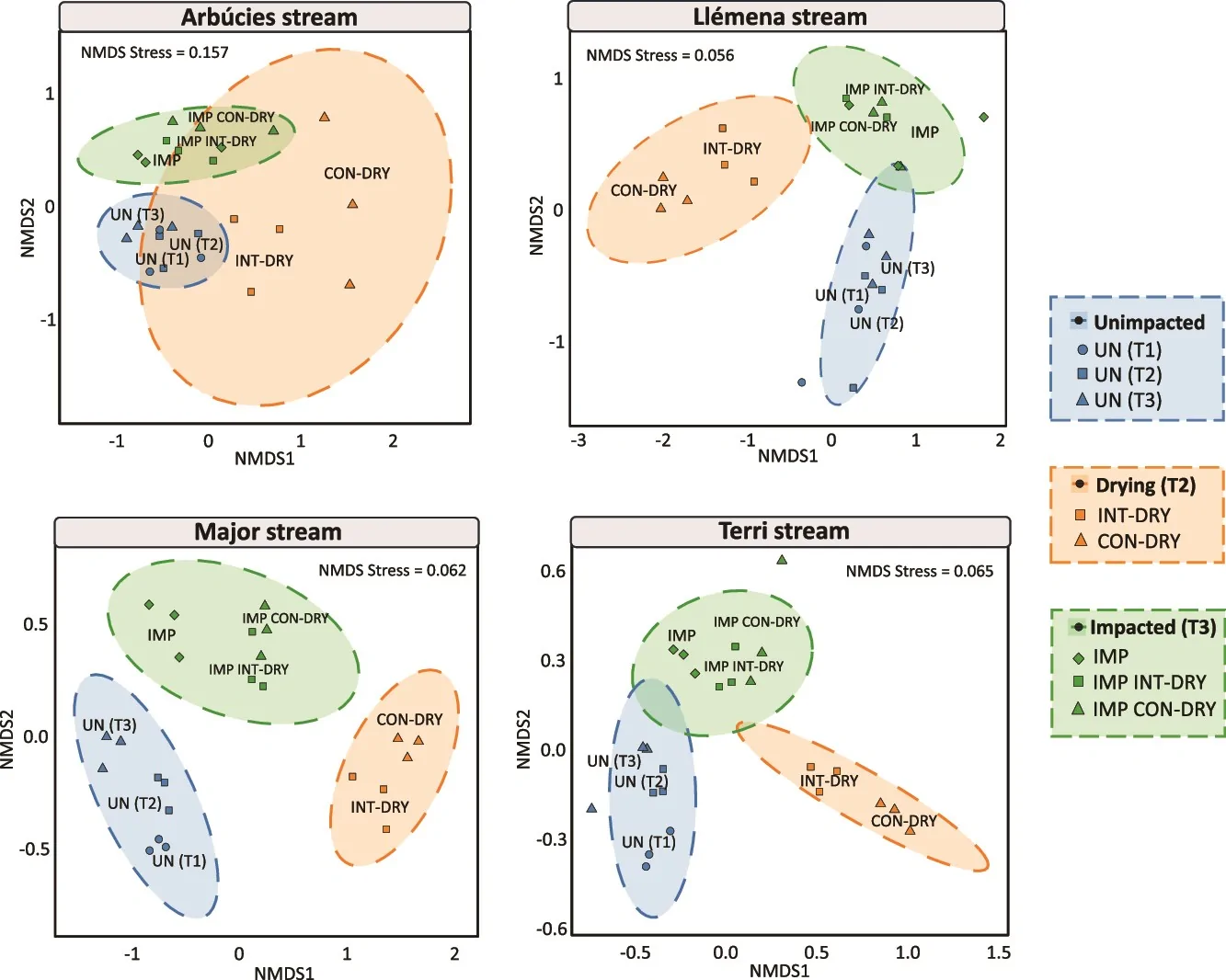
NMDS ordinations of biofilm bacterial community composition for each stream under unimpacted, drying, and impacted treatment blocks; different symbol shapes indicate treatments, and ellipses represent the 95% confidence interval for a multivariate distribution.

The largest shifts occurred between unimpacted and drying biofilms (*R*^2^ = 0.27–0.64), followed by drying vs impacted (*R*^2^ = 0.23–0.49), whereas differences between unimpacted and impacted biofilms were smaller (*R*^2^ = 0.28–0.39). Beta diversity partitioning showed that differences between treatment blocks were primarily driven by species turnover rather than nestedness. Turnover values were highest in Arbúcies, Major, and Llémena (β_SIM_ = 0.62–0.79), reflecting pronounced species replacement, and lowest in Terri (β_SIM_ = 0.41–0.60), indicating closer similarity between treatments. Nestedness contributed minimally to overall dissimilarity in all streams (β_SNE_ ≤ 0.14), suggesting that compositional differences were rarely explained by simple species loss or gain (Supplementary Table S8).

##### Regional beta diversity patterns

Beta diversity analysis showed consistent differences in biofilm community composition associated with treatment blocks, with some stream-specific variation. Drying caused the largest differentiation, while urban effluents produced smaller differences and greater overlap among streams (Fig. 5). PERMANOVA confirmed significant differences among streams (Supplementary Table S9), with Terri differing most strongly under both drying and effluent impacts (Supplementary Table S10).

**Figure 5:**
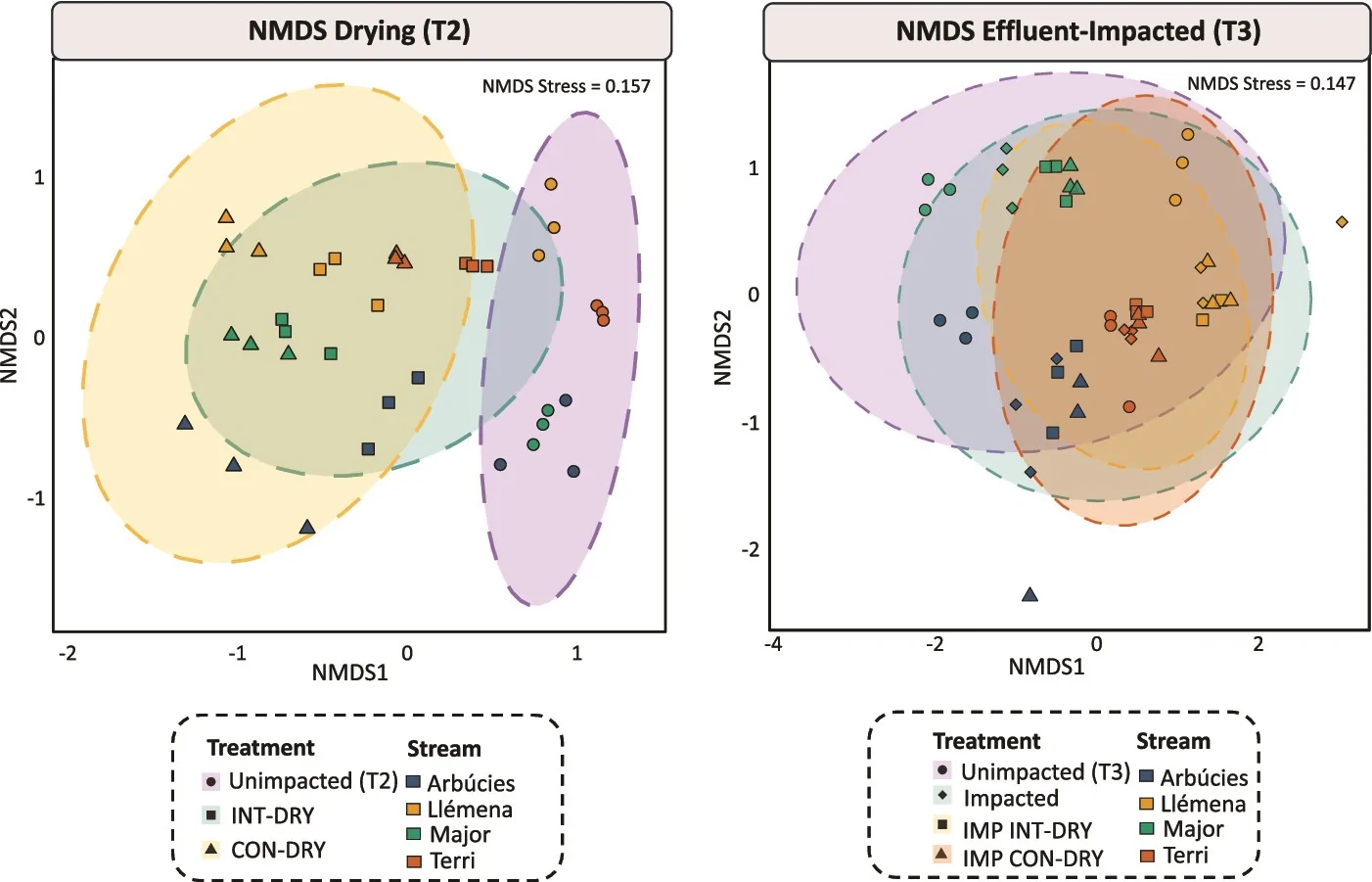
NMDS ordinations of biofilm bacterial community composition under drying treatments (T2) and effluent-impacted treatments (T3); points represent individual samples, with different symbol shapes indicating treatments and colors indicating streams; ellipses represent 95% confidence intervals around treatment centroids.

Drying significantly affected biofilm community composition, as shown by PERMANOVA and pairwise tests for each stream (*P* ≤ .001; Supplementary Table S9). Significant differences were observed among unimpacted (UN-T2), INT-DRY, and CON-DRY treatments across all streams, with pairwise comparisons explaining 10%–25% of the variation (Supplementary Table S10). Beta diversity partitioning indicated that these differences were mainly driven by taxa turnover rather than nestedness. In general, dissimilarity between unimpacted biofilms and those subjected to CON-DRY was consistently higher than with INT-DRY, reflecting a stronger compositional shift under CON-DRY (Supplementary Table S11).

Biofilms translocated to effluent-impacted reaches showed significant changes in bacterial community composition (PERMANOVA, *P* ≤ .001; Supplementary Table S9). The strongest differences (11%–12% of the variation; Supplementary Table S10) were observed between unimpacted biofilms (UN-T3) and those in impacted reaches previously exposed to drying (IMP-CON-DRY and IMP-INT-DRY). Differences among treatments within the impacted reach (IMP, IMP-CON-DRY, IMP-INT-DRY) were also significant but smaller (3%–6% of variation; Supplementary Table S10). Beta diversity decomposition indicated that these compositional shifts were primarily driven by taxa turnover, especially between unimpacted and impacted biofilms previously subjected to drying (Supplementary Table S12).

Considering both drying and urban effluents, prior drying homogenized the compositional response of biofilm communities to urban inputs. This convergence was evident in the NMDS ordination (Supplementary Fig. S5), with strong overlap between IMP INT-DRY and IMP CON-DRY, and no significant differences (PERMANOVA, *P* = .75; Supplementary Table S13). In contrast, communities only exposed to drying before translocation to effluent-impacted reaches showed higher dissimilarities, largely driven by taxa turnover, and were consistently greater under CON-DRY (CON-DRY–IMP CON-DRY) than under INT-DRY (INT-DRY–IMP INT-DRY; Supplementary Table S14).

### Key taxa driving stream community shifts

A recurrent set of families contributed disproportionately to the differences between the treatment blocks across the four stream biofilms (Fig. 6, Supplementary Table S15). Overall, 10–22 families accounted for 70% of the Bray–Curtis dissimilarity, indicating that community shifts were driven by a relatively small subset of taxa rather than by broad community changes.

**Figure 6:**
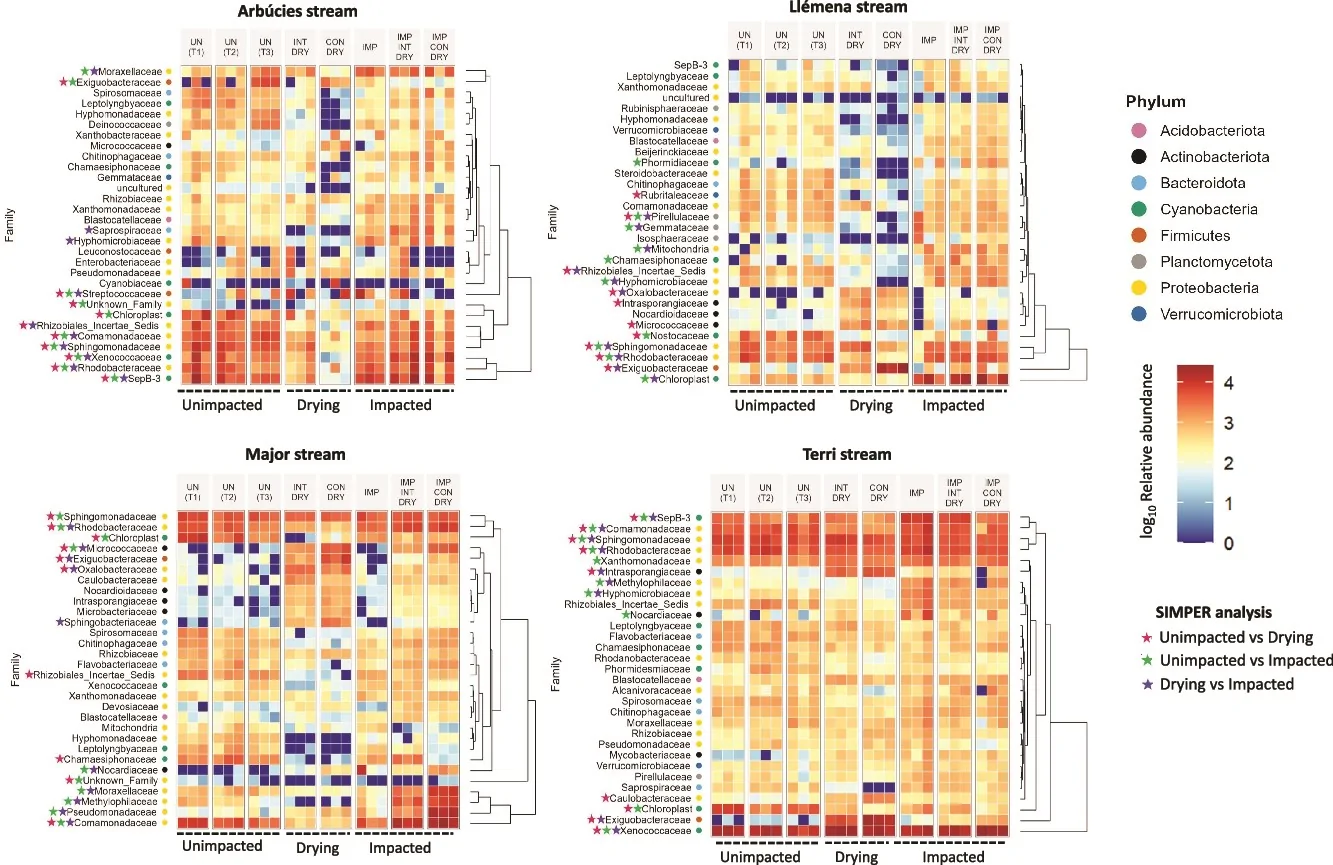
Community patterns at the family level and key taxa identified by SIMPER analysis among treatment blocks in each biofilm community; the heatmap shows log₁₀(relative abundance +1) of the 30 most abundant bacterial families across samples grouped by the eight experimental treatments: unimpacted (UN T1, UN T2, UN T3), drying (INT-DRY, CON-DRY), and impacted (IMP, IMP INT-DRY, IMP CON-DRY); families were ordered using hierarchical clustering based on their abundance profiles, and dots next to each family name indicate their phylum; stars mark families identified among the top 10 contributors by SIMPER to Bray–Curtis dissimilarity in each block-wise comparison (unimpacted–drying, unimpacted–impacted, and drying–impacted), and star color indicates the corresponding comparison.

Transitions from unimpacted to drying conditions were associated with declines in the relative abundance of several of the dominant bacterial families in unimpacted biofilms, including Sphingomonadaceae, Rhodobacteraceae, Comamonadaceae, and Rhizobiales. Cyanobacterial families also decreased, such as Leptolyngbyaceae (Arbúcies), Nostocaceae (Llémena), Leptolyngbyaceae and Chamaesiphonaceae (Major), and chloroplast-associated sequences (“Chloroplast”) in Terri. In contrast, drying conditions resulted in increases in the relative abundance of Exiguobacteraceae, Micrococcaceae, Intrasporangiaceae, Nocardioidaceae, and Oxalobacteraceae.

Subsequent translocation of biofilms to urban-impacted sites promoted bacterial families associated with wastewater. Moraxellaceae, Hyphomicrobiaceae, Methylophilaceae, and Nocardioidaceae increased in all streams, while others were stream-specific: Saprospiraceae (Arbúcies), Phormidiaceae and Chamaesiphonaceae (Llémena), and Pseudomonadaceae (Major).

Previous exposure to drying did not cause generalized differences in the bacterial community of translocated biofilms, except in Major, where stress-tolerant families that had increased during drying persisted and continued to contribute significantly to community dissimilarity, reflecting a legacy effect of the drying period (Fig. 6, Supplementary Table S15).

## Discussion

Drying and urban effluents significantly influenced the diversity and composition of bacterial biofilm communities across the four streams. Drying intensity, particularly during prolonged and CON-DRY, acted as the dominant environmental filter, driving pronounced taxonomic turnover, favoring the emergence of stress-tolerant taxa, but substantially reducing bacterial diversity. In contrast, urban effluents promoted community homogenization and the establishment of wastewater-associated taxa, overall resulting in increased richness. Notably, the sequential occurrence of drying followed by exposure to urban effluents produced a weak legacy on the composition of the bacterial community, with the magnitude of this change depending on local environmental conditions and the preexisting community composition in each biofilm.

### Drying intensity affects bacterial biofilm communities

Drying restructured the composition of bacterial biofilm communities, with the extent of change depending on the intensity of drying. INT-DRY caused moderate, transitional compositional changes, whereas CON-DRY triggered abrupt shifts in diversity and composition. CON-DRY exerted strong selective pressure, reducing richness, diversity, and evenness, increasing community dissimilarity, and promoting species turnover [39]. The predominance of turnover over nestedness indicates that drying actively reorganized community composition through the replacement of sensitive taxa by stress-tolerant species, reflecting deterministic assembly under sustained hydrological stress [51, 52]. In contrast, INT-DRY acted as a transitional disturbance, inducing compositional shifts without drastic community changes. This pattern is consistent with the idea that communities exposed to drying–rewetting cycles are shaped by disturbance history, allowing the persistence of multiple ecological strategies [36]. Alternating drying and rehydration phases may therefore allow less tolerant taxa to persist alongside resistant ones, facilitating rapid post-rewetting recovery [53] and maintaining ecosystem functions under fluctuating hydrological conditions [20, 21]. The effects also differed among streams: the strongest restructure occurred in biofilms of the Llémena stream, while the greatest resistance to drying was in the Terri stream biofilms, which may suggest a more initially stress-tolerant community in this stream. These findings highlight that drying intensity, frequency, and the intrinsic environmental characteristics of each stream are drivers of the microbial dynamics and structure [54–56].

Community-level dynamics under drying were dominated by taxa from the classes Bacilli and Actinobacteria, which are capable of rapidly colonizing streambed substrata under drying conditions [18]. These groups possess protective mechanisms such as endospore formation and the maintenance of cells in latent states [57, 58]. Within Bacilli, the family Exiguobacteraceae was particularly prominent under CON-DRY, reflecting its ability to persist in extreme and variable environments [59, 60]. Other families associated with drying, including Intrasporangiaceae, Micrococcaceae, and Nocardioidaceae, were also abundant, highlighting the adaptations of Actinobacteriota to harsh environments, such as filamentous structures and the production of small organic molecules that promote water retention and protect cellular functions during drying [61, 62]. In contrast, the cyanobacterial families Leptolyngbyaceae, Nostocaceae, and Chamaesiphonaceae consistently declined under drying, despite their ability to produce resistant structures and extracellular mucilage that aid water retention [63]. This decline was likely caused by competitive exclusion from stress-tolerant taxa. Under INT-DRY, early reactivation of resilient taxa may account for their re-occupation of suitable habitats [64] and the subsequent limitation of less tolerant species. Together, these dynamics indicate that both drying stress and competition regulate cyanobacterial persistence in biofilm communities submitted to this stressor.

### Impact of urban effluents on stream biofilms

Urban effluents promoted recolonization of biofilm bacterial communities, increasing the arrival of effluent-derived taxa and overall bacterial richness, indicating that WWTP effluents may substantially reshape community assembly in streams [22, 24]. Many ASVs in effluent-impacted biofilms were shared with those from unimpacted biofilms, reflecting both the effluent colonizers and the preexisting local community. Changes in translocated biofilms were dominated by species turnover rather than species loss, demonstrating that communities reorganize rather than lose taxa randomly [38]. Translocated biofilms in each stream had close community composition regardless of their previous history, illustrating the strong biotic homogenizing effect of urban effluents [65]. Further, some specific responses emerged in each stream as a result of their respective effluent characteristics.

Bacterial taxa introduced by urban effluents were mainly contaminant-tolerant and potentially pathogenic Gammaproteobacteria, including *Acinetobacter* (Moraxellaceae), *Pseudomonas* (Pseudomonadaceae), Methylophilaceae, and other families common in untreated and treated wastewaters [27, 66, 67]. These taxa are copiotrophic, metabolically versatile, and often harbor antibiotic resistance genes, giving them a competitive advantage in nutrient-rich environments [66, 68]. Saprospiraceae and Hyphomicrobiaceae also increased under effluent exposure, likely supporting enhanced organic matter degradation and nitrogen cycling [67, 69]. These compositional shifts were particularly pronounced in biofilms from Arbúcies and Major, likely due to higher nutrient and organic matter loads compared to their respective upstream sites [23]. Responses of phototrophic taxa were context-dependent in each stream. Cyanobacteria decreased in Arbúcies and Major, possibly reflecting the effects of reduced water transparency from turbidity and suspended solids [22, 70], whereas Phormidiaceae and Chamaesiphonaceae increased in Llémena, likely due to less damaging chemical conditions and potentially higher light availability in more open stretches, which may have favored phototrophic growth [71].

Overall, these results show that urban effluents reshape biofilm communities by favoring copiotrophic and contaminant-tolerant bacteria, while the abundance of photosynthetic taxa remains largely determined by local environmental conditions. These processes promoted species turnover and led to the homogenization of bacterial communities across streams, without necessarily restoring the original community composition.

### Combined effects of drying and urban effluents on biofilms

Drying and urban effluents have distinct effects on bacterial communities. While drying acts as a strong environmental filter, reducing diversity and favoring stress-tolerant taxa, exposure to urban effluents introduces new microorganisms and promotes recolonization. Their interaction creates a history of disturbance that can uniquely shape biofilm communities [72], as legacy effects from one stressor may influence responses to subsequent ones. In urban rivers affected by water abstraction or dams, dry streambeds may be intermittently replenished by treated effluents, whose nutrients and bacterial inocula can partially replace drying-resistant taxa and alter biomass and composition [24, 73]. However, this replacement is context-dependent and may be influenced both by the reach’s dilution capacity [74] and by the preexisting community.

Our observations confirmed that urban effluents act as strong vectors of microbial colonization, rapidly restructuring communities previously affected by drying [23, 71]. In particular, CON-DRY reduced bacterial diversity and richness, creating empty spaces for the secondary colonization by opportunistic, effluent-derived microorganisms, while reinforcing stress-tolerance mechanisms within the resident community [75]. Although INT-DRY initially caused milder changes, effluent inputs were sufficient to homogenize the resulting community composition [65]. Overall, effluent inputs largely overwhelmed the effects of prior disturbance legacies, indicating that drying effects appear mostly transient under strong urban influence [34]. Together, these patterns illustrate how environmental history and habitat modification shape microbial community resilience and assembly trajectories [72].

Despite the generally weak legacy of drying, some stress-tolerant taxa persisted after urban effluent exposure such as Actinobacteria. This was evident in the Major stream, where families such as Intrasporangiaceae, Micrococcaceae, and Nocardioidaceae were abundant during drying and persisted despite the influx of effluent-derived microorganisms. This pattern reflects a preexisting community in which only a few drying-tolerant taxa persisted, while most remained sensitive [33], highlighting a priority effect. Early colonizers during drying gain a competitive advantage and leave a legacy mediated by functional traits, such as resource transport, complex carbon degradation, and stress tolerance, which influence both resilience and the trajectory of microbial succession [64, 76]. Initial community composition also influenced responses to sequential stressors. The Major stream, a relatively pristine community characterized by low diversity, showed pronounced compositional shifts following disturbance, likely reflecting lower functional redundancy and limited metabolic plasticity [72, 77]. Such low initial complexity likely made the community more vulnerable and less flexible to desiccation, favoring stress-tolerant taxa that persist thereafter [36]. In contrast, more diverse communities from more urbanized streams, such as the Terri, exhibited greater resilience, probably due to broader functional and phylogenetic diversity, with contemporary effluent inputs playing a stronger role in shaping community composition.

Overall, these patterns indicate that environmental history and preexisting community structure modulate the intensity of legacy effects [78], determining whether sequential disturbances amplify or decrease community reorganization [79]. Although observations derive from four different systems, our findings suggest that bacterial communities from less impacted environments may be more sensitive to acute perturbations, while communities with a history of stress maintain greater structural and functional stability and are adapted to these occurring stressors [80, 81].

## Conclusions

Collectively, these findings underscore the importance of considering the sequence and intensity of disturbances to understand the dynamics of river biofilm communities exposed to multiple stressors. Drying acts as a strong environmental filter, with its intensity determining community restructuring: Bacilli and Actinobacteria dominate under CON-DRY, while Cyanobacteria are the most sensitive taxa at both levels of drying. Exposure to urban effluents promotes recolonization by effluent-derived taxa (e.g. Gammaproteobacteria), driving species turnover and leading to community homogenization within streams. The interaction between both stressors suggests that the legacy effects of drying are generally weak and often overridden by effluent inputs, jointly shaping community assembly in ways that cannot be predicted from single stressors. Furthermore, the environmental history and initial community composition modulate resistance and recovery to new pressures. Together, these results highlight the importance of ecological memory, taxon-specific responses, and colonization dynamics in determining microbial responses to global change, with important implications for predicting and managing the resilience of stream biofilm communities under increasing anthropogenic pressure.

## Additional information

Additional supporting information can be found online in the Supporting Information section.

## Supplementary Material

Supplementarymaterial_Effluentsoverridedryinginbiofilms_ycag270

## Data Availability

The data sets generated in this study are available in the NCBI Sequence Read Archive under BioProject accession number PRJNA1435015 at the following DOI: http://www.ncbi.nlm.nih.gov/bioproject/1435015
