# Supplementary material for: Urban effluents override drying legacy in stream biofilm bacterial communities
Source: ISME Commun. 2026 Sep 23;6(1):ycag270. doi: 10.1093/ismeco/ycag270 (PMC13653610; doi:10.1093/ismeco/ycag270)
Supplement: Supplementary file 1 [file supplementarymaterial_effluentsoverridedryinginbiofilms_ycag270.pdf]

SUPPLEMENTARY MATERIAL for article:

Urban effluents override drying legacy in stream biofilm bacterial communities

Javier Ortiz-Rivero[a, b] (jortiz@icra.cat)

Paula Siñeriz[a] (sinerizp@gmail.com)

Sergi Sabater[a,c] (ssabater@icra.cat)

Anna Freixa[a] (afreixa@icra.cat)

[a] Catalan Institute for Water Research (ICRA-CERCA), Girona, Carrer Emili Grahit 101, 17003 Girona, Spain.

[b] University of Girona, Plaça de Sant Domènec 3, 17004, Girona, Spain.

[c] Institute of Aquatic Ecology, University of Girona, Campus de Montilivi, 17071 Girona, Spain

Author for correspondence: Javier Ortiz-Rivero

Appendix S1: 2.5. Microbial diversity and composition analyses

2.5.1. Data preprocessing and rarefaction

Rarefaction curves were generated for each sample to evaluate whether the sequencing depth was sufficient to capture bacterial ASV richness. Adequate coverage was observed for all 96 samples, except for sample L23 (Llémena IMP INT-DRY-C), which exhibited very low sequencing depth (484 reads) and was therefore excluded from diversity analyses to avoid underestimation **(Figure S2)**. The remaining 95 samples yielded a total of 2,988 bacterial ASVs from 4,844,699 reads, with an average of 50,996 reads per sample (min–max: 8,601–135,513). For alpha diversity analyses, the ASV table was rarefied to 8,601 reads to ensure comparability across samples.

2.5.2. Bacterial composition and alpha diversity metrics

Bacterial composition

The taxonomic composition of bacterial communities was assessed at the class level using relative abundances calculated with the R package *phyloseq* [1]. Read counts in each sample were normalized to the mean sequencing depth, and the ten most abundant classes were retained, with all remaining lower-abundance taxa aggregated into a single category labelled "Others". These analyses were performed independently for each stream. Differences in the relative abundance of the main bacterial classes among the three treatment blocks (Unimpacted, Drying, Impacted) were assessed independently for each stream using Kruskal–Wallis tests, followed by Dunn's post hoc pairwise comparisons with Benjamini–Hochberg correction for multiple testing.

Alpha diversity metrics

ASV richness, Shannon index (to evaluate diversity), and Pielou's evenness (to assess community uniformity), were estimated from rarefied ASV tables (8601 reads) using *phyloseq* R package. Pielou's evenness was calculated as the Shannon index divided by the natural logarithm of ASV richness. Differences among treatments for each index were assessed using analysis of variance (ANOVA) with the *aov* function. When the assumptions of normality (Shapiro-Wilk test) or

homoscedasticity (Levene test) were not met, a non-parametric Kruskal–Wallis test was applied. Pairwise comparisons were performed using Tukey's honestly significant difference (HSD) test following ANOVA and Dunn's test with Bonferroni correction following Kruskal–Wallis. Data visualization were created using the R package *ggplot2* [2], and statistical results were integrated into the plots with *ggpubr* [3].

2.5.3. Beta diversity and distance-based redundancy (dbRDA) analysis

Beta diversity was performed using the ASV table normalized by the cumulative sum scaling (CSS) method, which corrects for differences in sequencing depth between samples [4]. Analyses were conducted at the local scale (within each stream, to test treatment effects) and regional scale (across all streams, to identify shared patterns). Non-metric multidimensional scaling (NMDS) based on Bray–Curtis dissimilarities was used to visualize differences in bacterial community composition across treatments at both scales, with ordination plots generated using *phyloseq* package.

At the local-scale, we estimated the differences in composition between treatment blocks (unimpacted, drying, and impacted) within each stream using Analysis of Similarities (ANOSIM) [5] and permutational multivariate analysis of variance (PERMANOVA) via the adonis function [6] with 999 permutations, implemented using the *vegan* R package [7]. At the regional scale, spatial structuring among biofilm communities was first evaluated by including stream as a factor in PERMANOVA. Treatment effects (drying and WWTP exposure) were then assessed by restricting permutations within each stream (option strata) followed by post-hoc pairwise test comparisons when significant.

To explore the sources of compositional dissimilarity, beta diversity was decomposed using the *betapart* package [8]. Total dissimilarity between groups and treatments was quantified using the Sørensen index (β_{SOR}), which ranges from 0 (identical communities) to 1 (completely different communities), species turnover based on ASV substitution (β_{SIM}), and nestedness resulting from

ASV loss (β_{NES}). This allowed differentiation when community changes were mainly driven by species replacement or by nested loss of taxa.

To evaluate the influence of environmental variables on bacterial community composition, a distance-based redundancy analysis (dbRDA) [9] was performed, applied exclusively to biofilm from unimpacted reaches of each stream. Analyses were based on Bray–Curtis dissimilarities of ASV abundances and were conducted using *vegan*. Environmental variables were standardized using z-score transformation to ensure their comparability, and those with high multicollinearity (VIF > 10) were excluded. The final dbRDA model was constructed with uncorrelated variables, and the significance of the axes and environmental predictors was assessed using ANOVA based on 500 permutations.

2.5.4. Community overlaps visualization and key taxa analysis

Shared and unique ASVs

Venn diagrams were generated independently for each stream using the *VennDiagram* package [10] to visualize the occurrence of each taxonomic unit (ASVs) shared or unique among the treatment blocks **(Table 1)**. Additionally, an UpSet plot was created using the *UpSetR* package [11] to examine exclusive or shared ASVs among these groups at the regional scale.

Identification of key dominant taxa

To identify the bacterial families driving the main compositional shifts, relative-abundance patterns were visualized using heatmaps of $\log_{10}$(abundance + 1) across the three treatment blocks (unimpacted, drying, and impacted). Heatmap columns corresponded to individual samples and were grouped by treatment. For clarity, the 30 most abundant families were selected in each stream based on cumulative relative abundance across all samples, the analysis being performed separately for each stream. In parallel, a SIMPER (Similarity Percentage) analysis was performed using the *vegan* R package to identify which taxa among the most abundant families contributed most to the compositional differences between treatment blocks (unimpacted-drying, unimpacted-impacted and drying-impacted). Taxa were ranked by their mean contribution to the Bray–Curtis

dissimilarity, and cumulative contributions explaining up to 70% of the total dissimilarity were retained. To facilitate visualization, the 10 taxa with the highest SIMPER contributions in each comparison were highlighted in the corresponding heatmap. SIMPER analyses were conducted independently for each stream.

References

1. McMurdie PJ, Holmes S. Phyloseq: an R package for reproducible interactive analysis and graphics of microbiome census data. *PLoS One* 2013;**8**:e61217. https://doi.org/10.1371/journal.pone.0061217

2. Wickham H. ggplot2. *Wiley Interdiscip Rev Comput Stat* 2011;**3**:180–85. https://doi.org/10.1002/wics.147

3. Kassambara A. ggpubr: 'ggplot2' Based Publication Ready Plots. R package version 0.6.3. 2026; https://10.32614/CRAN.package.ggpubr.

4. Paulson JN, Stine OC, Bravo HC et al. Differential abundance analysis for microbial marker-gene surveys. *Nat Methods* 2013;**10**:1200–2. https://doi.org/10.1038/nmeth.2658

5. Clarke KR. Non-parametric multivariate analyses of changes in community structure. *Austral J Ecol* 1993;**18**:117–43. https://doi.org/10.1111/j.1442-9993.1993.tb00438.x

6. Anderson MJ. A new method for non-parametric multivariate analysis of variance. *Austral Ecol* 2001;**26**:32–46. https://doi.org/10.1111/j.1442-9993.2001.01070.pp.x

7. Oksanen J et al. Vegan: community ecology package. R package version 2, 5–7. 2020; https://CRAN.R-project.org/package=vegan.

8. Baselga A, Orme CDL. *Betapart: an R package for the study of beta diversity*. *Methods Ecol Evol* 2012;**3**:808–12. https://doi.org/10.1111/j.2041-210X.2012.00224.x

9. Legendre P, Anderson MJ. Distance-based redundancy analysis: testing multispecies responses in multifactorial ecological experiments. *Ecol Monogr* 1999;**69**:1–24. https://doi.org/10.1890/0012-9615(1999)069[0001:DBRATM]2.0.CO;2

10. Chen H, Boutros PC. *VennDiagram*: a package for the generation of highly-customizable Venn and Euler diagrams in R. *BMC Bioinformatics* 2011;**12**:35. https://doi.org/10.1186/1471-2105-12-35

11. Conway JR, Lex A, Gehlenborg N. *UpSetR*: an R package for the visualization of intersecting sets and their properties. *Bioinformatics* 2017;**33**:2938–40. https://doi.org/10.1093/bioinformatics/btx364

Supplementary Figures

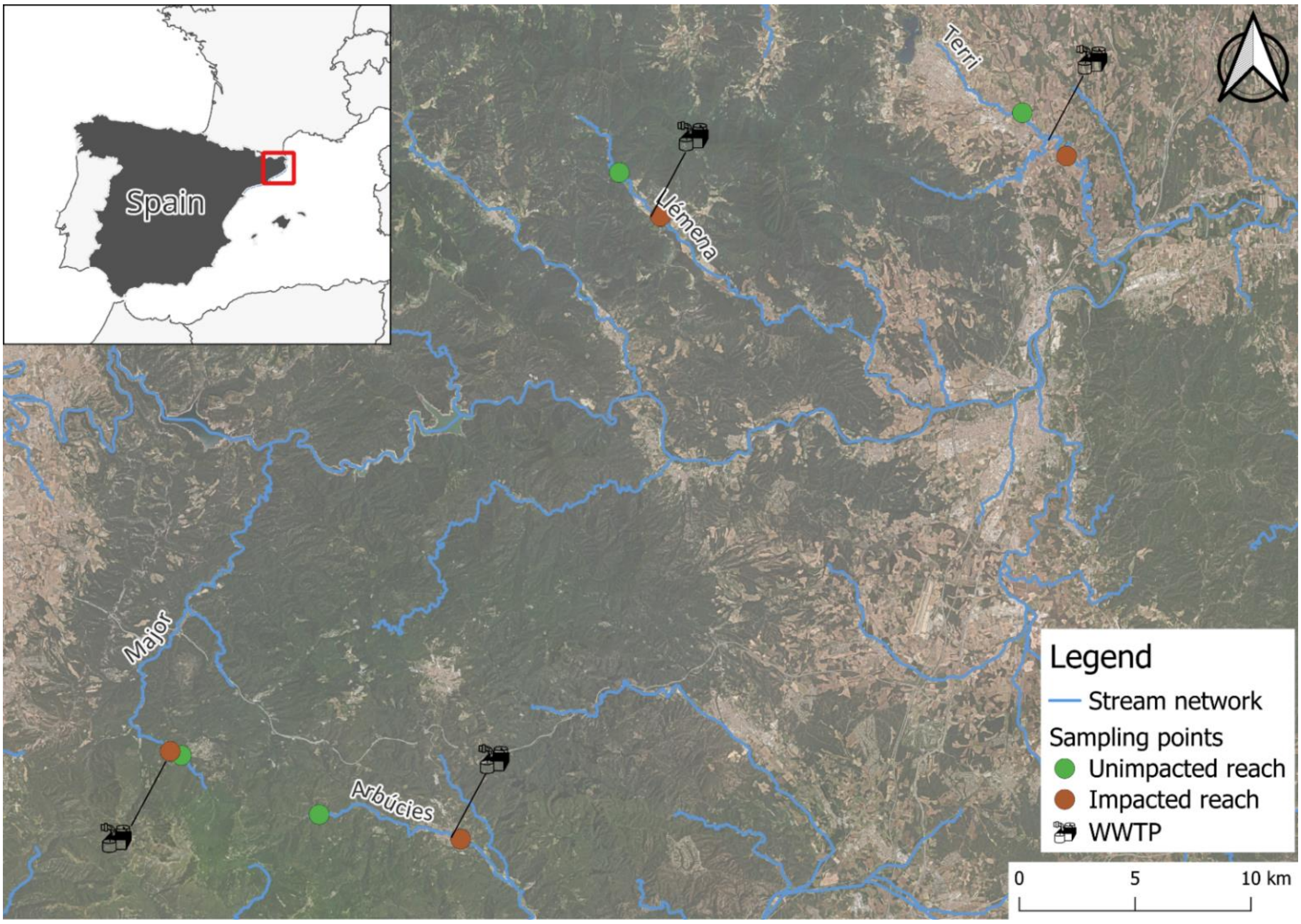

Figure S1. Map showing the location of the studied reaches in the streams Arbúcies, Llémena, Major, and Terri (green: unimpacted; brown: impacted) and their position in Girona, Spain.

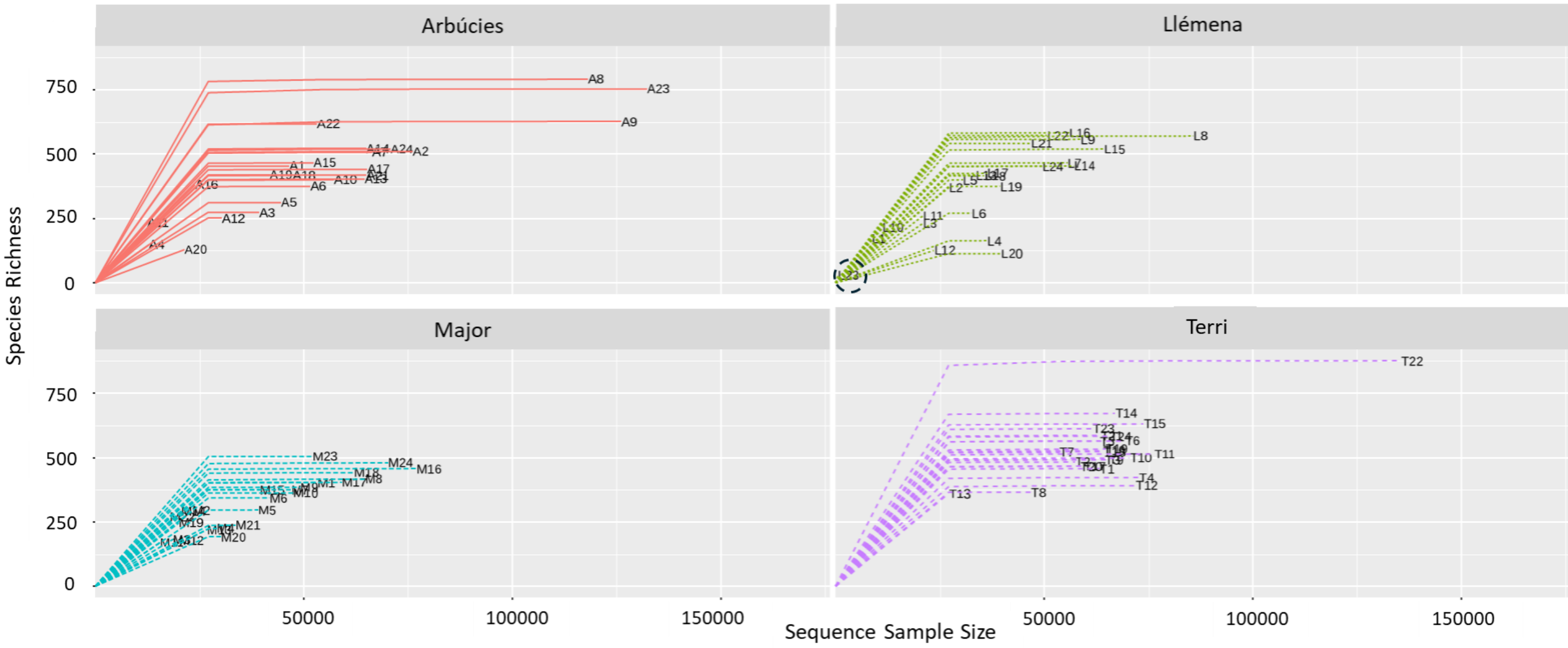

Figure S2. Rarefaction curves of bacterial amplicon sequence variant (ASV) richness in biofilm communities from different streams (Arbúcies, Llémena, Major, and Terri), including all samples from each stream across the different treatments.

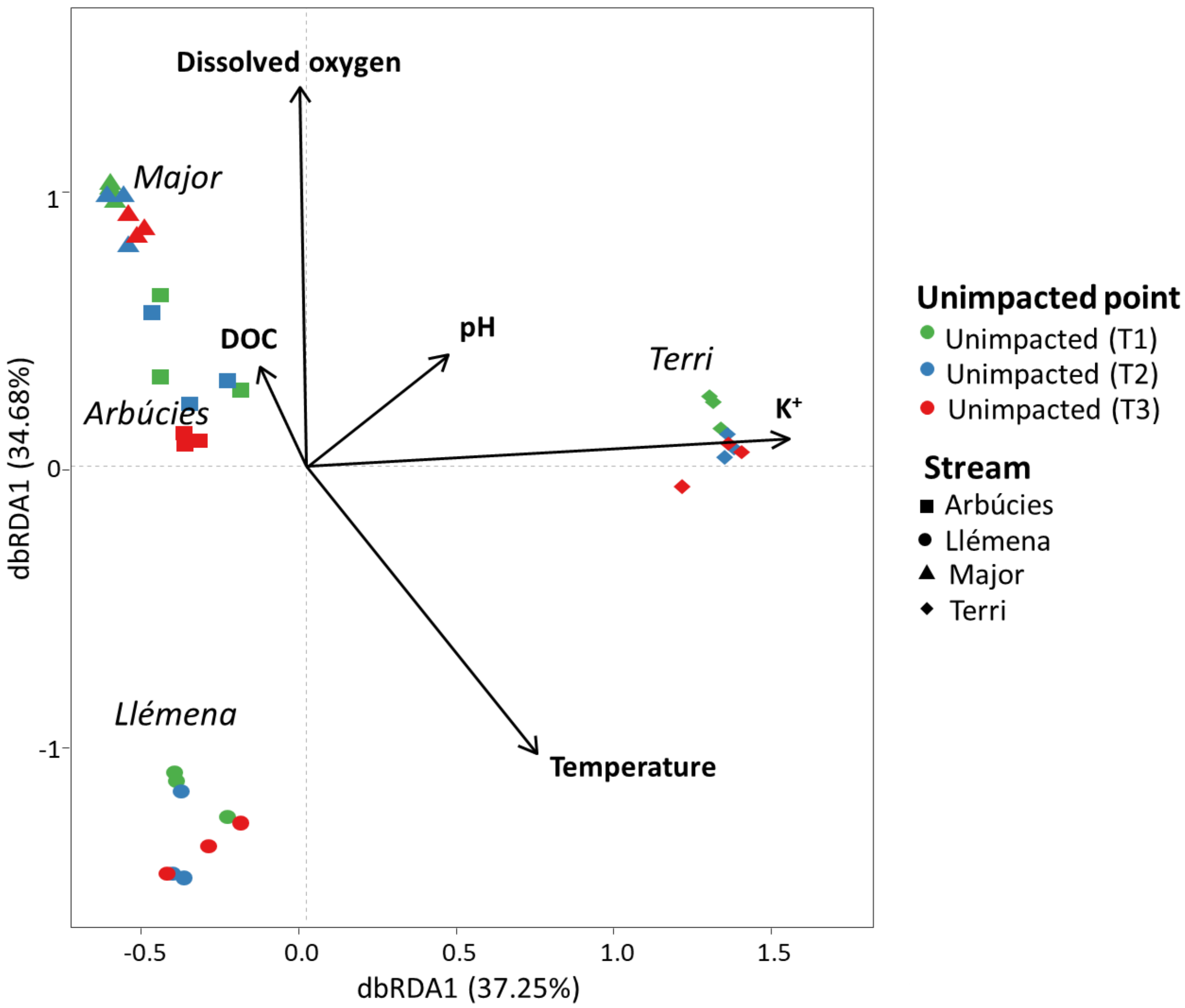

Figure S3. Distance-based redundancy analysis (db-RDA) showing the environmental variables that explained the greatest proportion of variability in bacterial biofilm communities across different streams (indicated by shape). Samples from each unimpacted stream are shown in different colors according to sampling time (T1, T2, and T3).

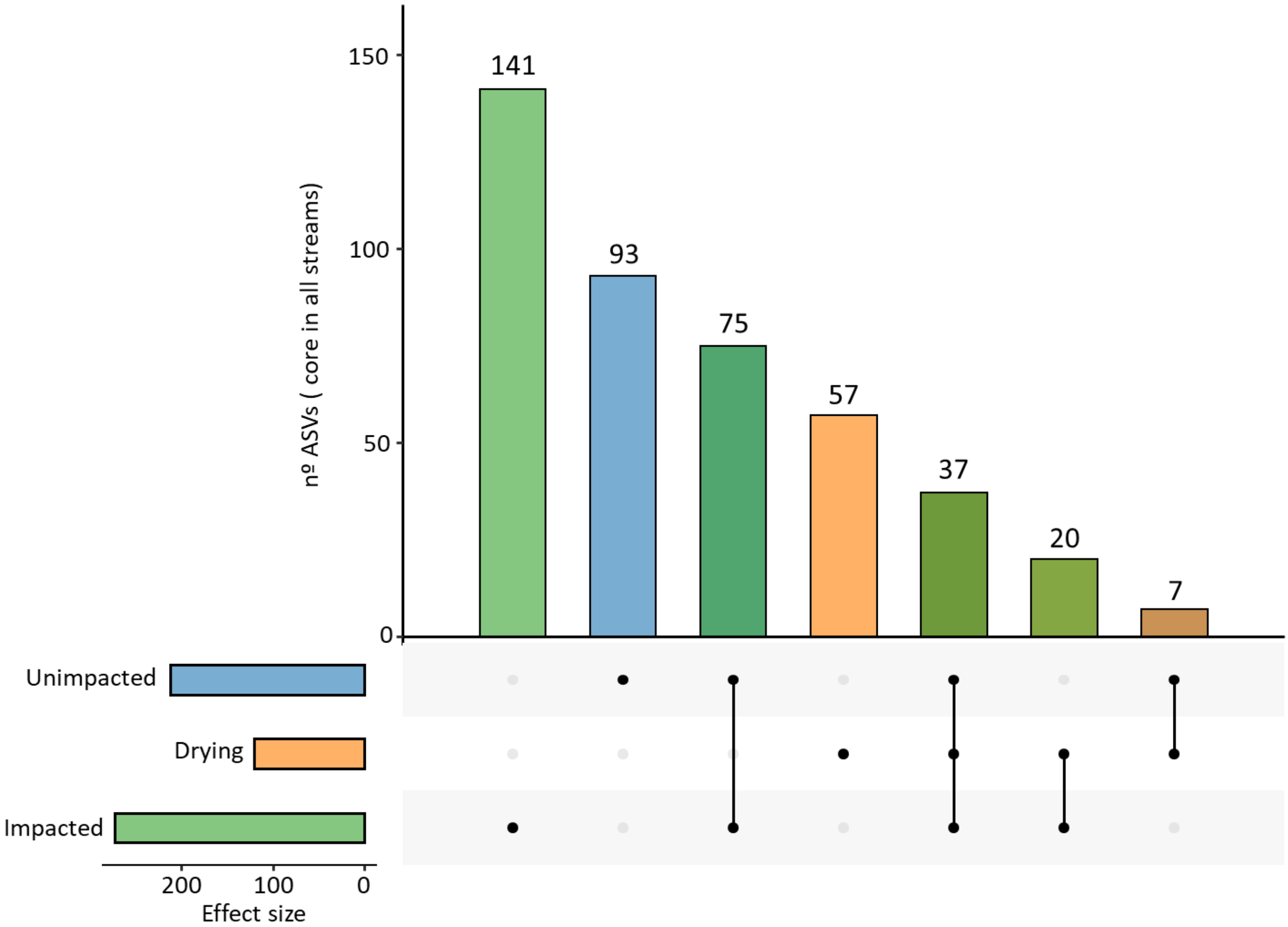

Figure S4. UpSet plot showing the number of bacterial ASVs consistently detected across all four streams under each condition (Unimpacted, Drying, and Impacted). Bars represent the size of each intersection set, indicating ASVs shared among conditions or exclusive to a single condition, considering only ASVs present in all four streams.

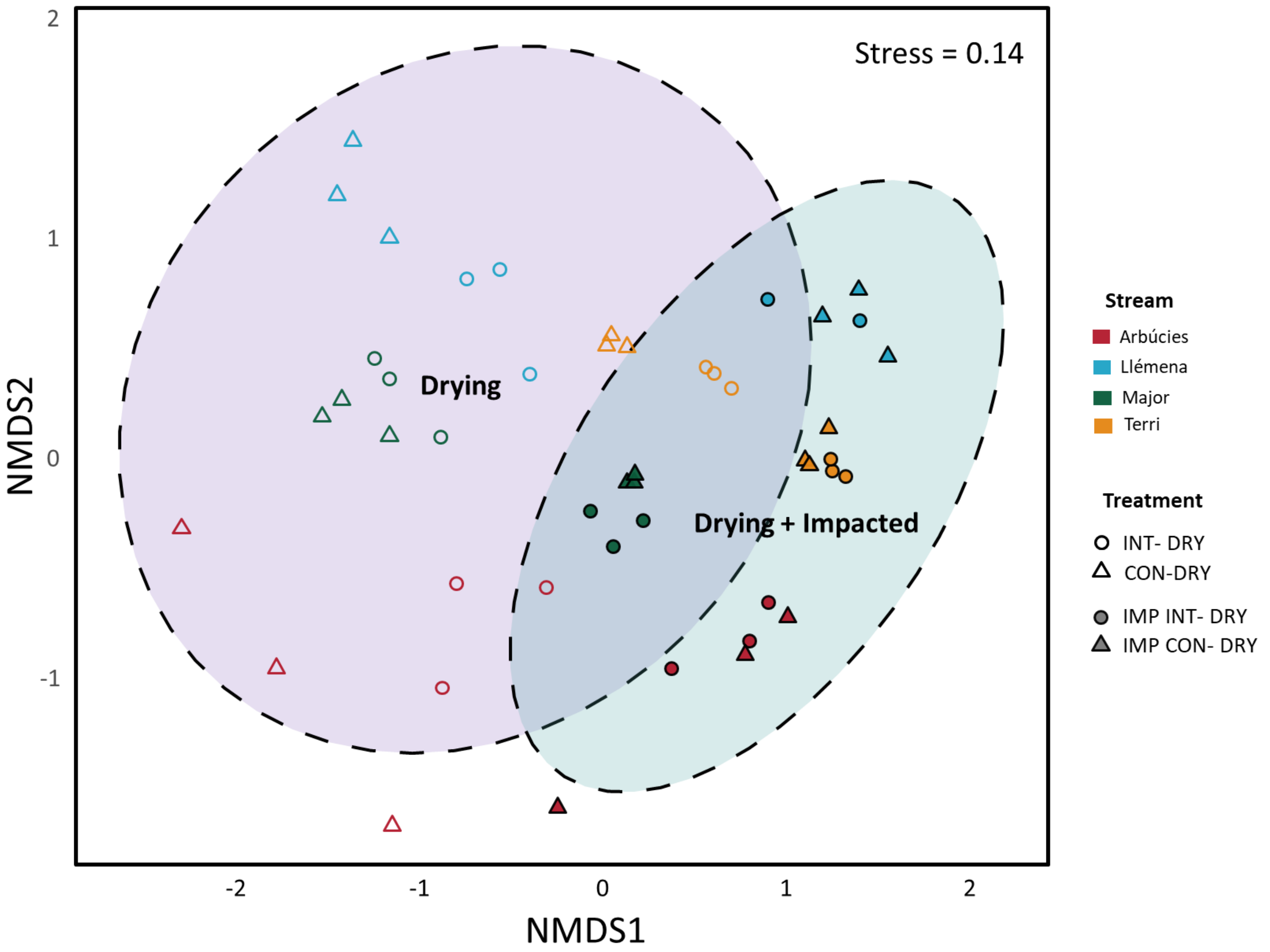

Figure S5. Non-metric multidimensional scaling (NMDS) ordination based on Bray-Curtis dissimilarities of bacterial biofilm communities across four streams, showing how drying may have legacy effects on community composition. Samples are grouped by treatment: "Drying" (purple) and "Drying + Impacted" (blue). Points are shaped according to base treatment (INT = circle, CON = triangle) and colored by stream (Arbúcies, Llémena, Major, Terri).

Supplementary Tables

Table S1. Physicochemical water parameters measured at the unimpacted reach of each stream (Arbúcies, Llémena, Major, and Terri) during three sampling periods (T1–T3), and at the impacted reach downstream of the WWTP during the final sampling period (T3). The limit of quantification (LOQ) for NO_2^-, PO_4^{3-} and NH_4^+ was 0.003 mg N–NO_2^- L^{-1}, 0.008 mg P–PO_4^{3-} L^{-1} and 0.004 mg N–NH_4^+ L^{-1}, respectively.

**UNIMPACTED REACH**					
**Variable**	**Treatment**	**Arbúcies**	**Llémena**	**Major**	**Terri**
**pH**	Unimpacted (T1)	7.75	8.07	7.95	8.18
	Unimpacted (T2)	7.95	7.78	8.14	7.90
	Unimpacted (T3)	7.22	7.53	7.80	7.93
**Temperature (ºC)**	Unimpacted (T1)	15.4	15.8	9.8	18.1
	Unimpacted (T2)	18.7	18.2	11.0	16.3
	Unimpacted (T3)	17.0	17.8	11.8	18.5
**Conductivity (µS cm^{-1})**	Unimpacted (T1)	236	594	240	1508
	Unimpacted (T2)	241	596	248	1512
	Unimpacted (T3)	232	549	226	1393
**Dissolved oxygen (mg L^{-1})**	Unimpacted (T1)	8.36	7.62	10.3	9.93
	Unimpacted (T2)	8.31	6.52	9.47	7.87
	Unimpacted (T3)	7.57	6.18	8.8	7.25
**Cl^- (mg L^{-1})**	Unimpacted (T1)	4.72	12.19	9.15	48.87
	Unimpacted (T2)	5.59	9.26	8.96	49.92
	Unimpacted (T3)	7.93	16.52	9.11	44.74
**SO_4^{2-} (mg L^{-1})**	Unimpacted (T1)	1.47	3.80	3.29	185.77
	Unimpacted (T2)	1.51	2.81	4.27	188.88
	Unimpacted (T3)	1.99	4.58	3.31	168.04
**Na^+ (mg L^{-1})**	Unimpacted (T1)	4.38	8.53	9.79	33.60
	Unimpacted (T2)	5.35	6.76	10.46	34.49
	Unimpacted (T3)	7.34	10.99	9.62	31.75
**K^+ (mg L^{-1})**	Unimpacted (T1)	1.06	1.07	1.09	3.29
	Unimpacted (T2)	1.32	1.34	1.14	3.46
	Unimpacted (T3)	1.58	1.01	1.05	3.37
**Mg^{2+} (mg L^{-1})**	Unimpacted (T1)	3.35	16.45	5.9	33.17
	Unimpacted (T2)	3.61	12.49	6.42	33.25
	Unimpacted (T3)	4.75	10.20	5.73	29.79
**Ca^{2+} (mg L^{-1})**	Unimpacted (T1)	16.02	30.17	25.01	232.44
	Unimpacted (T2)	19.13	28.60	24.73	241.89
	Unimpacted (T3)	24.28	25.67	24.35	220.37
**DOC (mg C L^{-1})**	Unimpacted (T1)	1.26	1.10	1.32	1.3
	Unimpacted (T2)	0.91	0.88	1.11	1.04
	Unimpacted (T3)	2.23	1.15	1.22	1.16
**NO_2^- (mg L^{-1})**	Unimpacted (T1)	<LOQ	<LOQ	0.004	0.082
	Unimpacted (T2)	<LOQ	<LOQ	0.011	0.120
	Unimpacted (T3)	<LOQ	0.004	0.004	0.161
**NO_3^- (mg L^{-1})**	Unimpacted (T1)	0.18	0.63	0.59	3.61
	Unimpacted (T2)	0.16	0.44	0.42	3.37
	Unimpacted (T3)	0.23	0.51	0.41	3.02
**NH_4^+ (mg L^{-1})**	Unimpacted (T1)	<LOQ	0.02	0.01	0.01
	Unimpacted (T2)	<LOQ	0.02	<LOQ	0.02
	Unimpacted (T3)	<LOQ	0.01	<LOQ	0.05
**PO_4^{3-} (mg L^{-1})**	Unimpacted (T1)	<LOQ	<LOQ	<LOQ	<LOQ

	Unimpacted (T2)	<LOQ	<LOQ	<LOQ	<LOQ
	Unimpacted (T3)	<LOQ	<LOQ	<LOQ	<LOQ
**IMPACTED REACH**					
**Variable**	Treatment	Arbúcies	Llémena	Major	Terri
**pH**	Impacted (T3)	7.3	7.8	8.7	7.6
**Temperature (ºC)**	Impacted (T3)	22.1	22	15.1	19.6
**Conductivity (µS cm^{-1})**	Impacted (T3)	452	1022	1228	1575
**Dissolved oxygen (mg L^{-1})**	Impacted (T3)	5.9	6	7.4	4.7
**Cl^- (mg L^{-1})**	Impacted (T3)	62.6	195.4	72.1	113.9
**SO_4^{2-} (mg L^{-1})**	Impacted (T3)	9.5	10.8	36	138.2
**Na^+ (mg L^{-1})**	Impacted (T3)	43.3	123.8	225.1	75.4
**K^+ (mg L^{-1})**	Impacted (T3)	9.1	15.7	8	7.7
**Mg^{2+} (mg L^{-1})**	Impacted (T3)	7.7	18.8	7.3	23.8
**Ca^{2+} (mg L^{-1})**	Impacted (T3)	34.5	48.6	28.2	179.1
**DOC (mg C L^{-1})**	Impacted (T3)	7.9	3.9	5.2	4
**NO_2^- (mg L^{-1})**	Impacted (T3)	0.37	<LOQ	0.03	0.38
**NO_3^- (mg L^{-1})**	Impacted (T3)	0.91	0.32	0.61	3.3
**NH_4^+ (mg L^{-1})**	Impacted (T3)	0.44	0.03	0.38	0.30
**PO_4^{3-} (mg L^{-1})**	Impacted (T3)	0.44	<LOQ	0.63	0.33

Table S2. Results of permutation-based ANOVA tests assessing the significance of dbRDA axes and the contribution of selected physicochemical variables to variation in biofilm bacterial community structure at unimpacted stream reaches.

		**Df**	**Sum of Sqs.**	**F**	**Pr (> F)**
**Axes**	*dbRDA1*	1	2.27	14.15	0.001
	dbRDA2	1	2.11	13.18	0.001
	Residual	30	4.81		
**Environmental variables**	*pH*	1	0.93	5.79	0.001
	Temperature	1	1.79	11.15	0.001
	Dissolved oxygen	1	1.54	9.57	0.001
	K^+	1	1.55	9.67	0.001
	DOC	1	0.29	1.83	0.070
	Residual	30	4.81		

Table S3. Statistical comparison of the relative abundance of the dominant bacterial classes among treatment blocks within each stream. Overall differences were evaluated using Kruskal–Wallis tests, followed by Dunn's post hoc pairwise comparisons. Arrows indicate the direction of change in relative abundance from the first group to the second group of each comparison, inferred from the sign of the Dunn's test statistic (Z value), whereas asterisks indicate the adjusted significance level, whereas "ns" indicates non-significant differences. Only bacterial classes showing a significant overall Kruskal–Wallis test are included.

Stream	Bacterial class	p value	Unimpacted → Drying	Drying → Impacted
**Arbúcies**	Actinobacteria	0.0021 **	↑ ***	ns
**Llémena**	Actinobacteria	0.0395 *	↑ *	ns
	Alphaproteobacteria	0.0395 *	↓ *	ns
	Bacilli	0.0358 *	↑ *	↓ *
	Bacteroidia	0.0395 *	ns	ns
	Cyanobacteriia	0.0201 *	↓ *	↑ **
	Gammaproteobacteria	0.0201 *	ns	↓ *
	Planctomycetes	0.0395 *	ns	ns
	Verrucomicrobiae	0.0358 *	↓ *	ns
**Major**	Actinobacteria	0.0003 ***	↑ ****	ns
	Bacilli	0.0015 **	↑ ***	↓ *
	Cyanobacteriia	0.0003 ***	↓ ****	ns
	Gammaproteobacteria	0.0314 *	ns	ns
	Verrucomicrobiae	0.0128 *	↓ **	↑ *
**Terri**	Actinobacteria	0.0005 ***	↑ ****	↓ *
	Alphaproteobacteria	0.0193 *	↓ *	ns
	Bacilli	0.0007 ***	↑ ***	↓ *
	Bacteroidia	0.0013 **	↓ ***	↑ *
	Gammaproteobacteria	0.0060 **	↓ *	↑ **
	Planctomycetes	0.0007 ***	ns	↑ ***

Table S4. Percentage of bacterial ASVs exclusive to each condition (unimpacted, drying, impacted) and shared between conditions (U–D, U–I, D–I) or among all three conditions (U–D–I), for each stream. Percentages are calculated relative to the total ASVs per stream across all conditions. U = unimpacted; D = drying; I = impacted.

	**Exclusive ASVs (%)**			**Shared ASVs (%)**			
**Stream**	*Unimpacted (U)*	*Drying (D)*	*Impacted (I)*	*U–D*	*U-I*	*D–I*	*U–D–I*
Arbúcies	14.0	4.9	24.3	4.6	16.4	7.0	23.2
Llémena	21.7	9.3	24.2	7.8	28.4	5.2	17.7
Major	26.2	8.8	25.9	3.1	35.3	10.6	17.2
Terri	7.9	8.4	12.1	3.0	19.7	8.9	29.7

Table S5. Average alpha-diversity indices (Richness, Shannon, Pielou's evenness) of biofilm communities in each treatment and stream (Arbúcies, Llémena, Major, and Terri). Treatments correspond to unimpacted across different sampling periods (UN T1-T3), drying (INT-DRY, CONT-DRY) and impacted (IMP, IMP-INT DRY and IMT CON-DRY).

	**ARBÚCIES STREAM**			
	**Treatment**	**Richness**	**Shannon**	**Evenness**
**UNIMPACTED**	*UN (T1)*	460	4.90	0.80
	UN (T2)	413	4.94	0.82
	UN (T3)	354	4.65	0.79
**DRYING**	*INT-DRY (T2)*	304	4.62	0.81
	CONT-DRY (T2)	176	3.58	0.69
**IMPACTED**	*IMP (T3)*	480	4.90	0.79
	IMP INT-DRY (T3)	520	4.74	0.76
	IMP CON-DRY (T3)	520	5.11	0.82
	**LLÉMENA STREAM**			
**UNIMPACTED**	*UN (T1)*	375	5.03	0.87
	UN (T2)	330	4.92	0.86
	UN (T3)	442	5.14	0.84
**DRYING**	*INT-DRY (T2)*	283	4.66	0.83
	CONT-DRY (T2)	133	1.80	0.37
**IMPACTED**	*IMP (T3)*	411	4.60	0.77
	IMP INT-DRY (T3)	454	4.40	0.72
	IMP CON-DRY (T3)	501	4.65	0.75
	**MAJOR STREAM**			
**UNIMPACTED**	*UN (T1)*	373	4.85	0.82
	UN (T2)	351	4.62	0.79
	UN (T3)	246	4.28	0.78
**DRYING**	*INT-DRY (T2)*	200	4.33	0.82
	CONT-DRY (T2)	194	3.84	0.73
**IMPACTED**	*IMP (T3)*	295	4.78	0.84
	IMP INT-DRY (T3)	406	4.79	0.80
	IMP CON-DRY (T3)	439	4.57	0.75
	**TERRI STREAM**			
**UNIMPACTED**	*UN (T1)*	439	4.83	0.79
	UN (T2)	470	4.96	0.81
	UN (T3)	469	5.11	0.83
**DRYING**	*INT-DRY (T2)*	472	4.50	0.73
	CONT-DRY (T2)	392	4.18	0.70
**IMPACTED**	*IMP (T3)*	630	5.21	0.81
	IMP INT-DRY (T3)	554	4.93	0.78
	IMP CON-DRY (T3)	459	4.37	0.71

Table S6. ANOSIM and adonis test (PERMANOVA) results testing for differences in community composition among the three treatment blocks (unimpacted, drying, impacted) within each stream, based on Bray–Curtis dissimilarities. Higher R and R^2 values indicate greater dissimilarity between groups and p-value<0.001 significant differences.

Biofilm community	ANOSIM Global test		Adonis test	
	R	p-value	R^2	p-value
**Arbúcies**	0.66	0.001	0.32	0.001
**Llémena**	0.94	0.001	0.51	0.001
**Major**	0.89	0.001	0.55	0.001
**Terri**	0.88	0.001	0.53	0.001

Table S7. Pairwise PERMANOVA results (F, R^2, p value) for biofilm communities in the four streams, comparing unimpacted–drying, unimpacted–impacted, and drying–impacted treatment blocks.

Stream	Unimpacted-Drying			Unimpacted-Impacted			Drying-Impacted		
	F value	R^2	p-value	F value	R^2	p-value	F value	R^2	p-value
**Arbúcies**	4.88	0.27	<0.001	6.29	0.28	<0.001	3.91	0.23	<0.001
**Llémena**	11.25	0.47	<0.001	9.50	0.39	<0.001	10.46	0.46	<0.001
**Major**	22.72	0.63	<0.001	9.26	0.37	<0.001	10.31	0.44	<0.001
**Terri**	16.86	0.56	<0.001	8.33	0.34	<0.001	12.36	0.49	<0.001

Table S8. Pairwise compositional dissimilarities of biofilm communities in the Arbúcies, Llémena, Major, and Terri streams, expressed as Sørensen dissimilarity (β_{SOR}), turnover (β_{SIM}), and nestedness dissimilarity (β_{SNE}) among unimpacted, drying, and impacted treatment blocks.

Stream	Treatment block	βsor (Sorensen)	βsim (Turnover)	βsne (Nestedness)
**Arbúcies**	Unimpacted-Drying	0.79	0.70	0.09
	Unimpacted-Impacted	0.70	0.65	0.05
	Drying-Impacted	0.80	0.66	0.14
**Llémena**	Unimpacted-Drying	0.81	0.71	0.10
	Unimpacted-Impacted	0.69	0.62	0.07
	Drying-Impacted	0.82	0.68	0.14
**Major**	Unimpacted-Drying	0.85	0.79	0.06
	Unimpacted-Impacted	0.67	0.62	0.05
	Drying-Impacted	0.75	0.62	0.13
**Terri**	Unimpacted-Drying	0.62	0.60	0.03
	Unimpacted-Impacted	0.49	0.41	0.08
	Drying-Impacted	0.56	0.49	0.07

Table S9. PERMANOVA results testing the effects of stream and treatment on biofilm bacterial community composition under drying and wastewater exposure (impacted) conditions.

	Factor	SumOfSqs	R^2	F	Pr(>F)
**Drying**	Stream	4.18	0.34	7.70	1e-04
	Treatment	2.83	0.23	7.83	1e-04
	Residual	5.42	0.44		
**Impacted**	Stream	6.86	0.44	13.11	1e-04
	Treatment	1.69	0.11	3.23	1e-04
	Residual	6.98	0.45		

Table S10. Pairwise PERMANOVA results for biofilm bacterial community composition, showing F values, R^2, and p-values for comparisons between streams (Arbúcies, Llémena, Major and Terri) and treatments under drying (UN T2, CON-DRY, INT-DRY) and impacted (UN T3, IMP, IMP CON DRY, IMP INT DRY) conditions.

	Factor	Comparasion	F	R^2	p-value
**DRYING**	Stream	Arbúcies vs Llémena	3.44	0.18	0.0009
		Arbúcies vs Major	3.01	0.16	0.0103
		Arbúcies vs Terri	7.89	0.33	0.0001
		Llémena vs Major	3.31	0.17	0.0073
		Llémena vs Terri	7.91	0.33	0.0001
		Major vs Terri	9.78	0.38	0.0001
	Treatment	UN (T2) vs CON-DRY	7.32	0.25	1e-04
		UN (T2) vs INT-DRY	4.75	0.18	1e-04
		CON-DRY vs INT-DRY	2.42	0.10	1e-04
**IMPACTED**	Stream	Arbúcies vs Llémena	10.34	0.33	1e-04
		Arbúcies vs Major	7.91	0.26	1e-04
		Arbúcies vs Terri	14.39	0.40	1e-04
		Llémena vs Major	9.61	0.31	1e-04
		Llémena vs Terri	15.07	0.42	1e-04
		Major vs Terri	13.35	0.38	1e-04
	Treatment	UN (T3) vs IMP	1.45	0.06	0.0001
		UN (T3) vs IMP-CON DRY	3.05	0.12	0.0003
		UN (T3) vs IMP-INT DRY	2.72	0.11	0.0001
		IMP vs IMP-CON DRY	1.42	0.06	0.0008
		IMP vs IMP-INT DRY	1.24	0.06	0.0013
		IMP-CON DRY vs IMP INT-DRY	0.62	0.03	0.0267

Table S11. Pairwise compositional dissimilarities of biofilm communities in the Arbúcies, Llémena, Major, and Terri streams, expressed as Sørensen dissimilarity (β_{SOR}), turnover (β_{SIM}), and nestedness dissimilarity (β_{SNE}) among drying treatments (UN T2, CON-DRY, INT-DRY).

Stream	Group	β_{SOR} (Sørensen)	β_{SIM} (Turnover)	β_{SNE} (Nestedness)
**Arbúcies**	UN (T2)-CON DRY	0.86	0.74	0.12
	UN (T2)-INT DRY	0.70	0.62	0.08
**Llémena**	UN (T2)-CON DRY	0.86	0.76	0.11
	UN (T2)-INT DRY	0.78	0.74	0.04
**Major**	UN (T2)-CON DRY	0.84	0.77	0.07
	UN (T2)-INT DRY	0.80	0.71	0.09
**Terri**	UN (T2)-CON DRY	0.66	0.63	0.03
	UN (T2)-INT DRY	0.55	0.54	0.01

Table S12. Pairwise compositional dissimilarities of biofilm communities in the Arbúcies, Llémena, Major, and Terri streams, expressed as Sørensen dissimilarity (β_{SOR}), turnover (β_{SIM}), and nestedness dissimilarity (β_{SNE}) among effluent impacted treatments (UN T3, IMP, IMP CON DRY, IMP INT DRY).

Stream	Group	β_{SOR} (Sørensen)	β_{SIM} (Turnover)	β_{SNE} (Nestedness)
**Arbúcies**	UN (T3)-IMP	0.63	0.56	0.07
	UN (T3)-IMP CON DRY	0.76	0.69	0.07
	UN (T3)-IMP INT DRY	0.68	0.60	0.08
**Llémena**	UN (T3)-IMP	0.65	0.59	0.06
	UN (T3)-IMP CON DRY	0.63	0.59	0.04
	UN (T3)-IMP INT DRY	0.66	0.64	0.03
**Major**	UN (T3)-IMP	0.61	0.56	0.05
	UN (T3)-IMP CON DRY	0.75	0.65	0.10
	UN (T3)-IMP INT DRY	0.73	0.63	0.09
**Terri**	UN (T3)-IMP	0.43	0.31	0.13
	UN (T3)-IMP CON DRY	0.54	0.48	0.06
	UN (T3)-IMP INT DRY	0.48	0.41	0.07

Table S13. Pairwise PERMANOVA results for biofilm bacterial community composition, showing F values, R^2, and p-values for comparisons between streams (Arbúcies, Llémena, Major and Terri) and treatments, including biofilms exposed to drying (CON-DRY, INT-DRY) and biofilms exposed sequentially to drying followed by WWTP effluent impacted (IMP CON-DRY, IMP INT-DRY).

Factor	Comparasion	F	R^2	p-value
Stream	Arbúcies vs Llémena	4.72	0.18	1e-04
	Arbúcies vs Major	5.31	0.19	1e-04
	Arbúcies vs Terri	10.54	0.32	1e-04
	Llémena vs Major	5.48	0.21	4e-04
	Llémena vs Terri	11.35	0.35	1e-04
	Major vs Terri	15.52	0.41	1e-04
Treatment	CON-DRY vs IMP CON-DRY	5.30	0.19	0.0001
	CON-DRY vs INT-DRY	2.42	0.10	0.0169
	CON-DRY vs IMP INT-DRY	5.98	0.22	0.0001
	IMP CON-DRY vs INT-DRY	3.56	0.14	0.0005
	IMP CON-DRY vs IMP INT-DRY	0.62	0.03	0.7486
	INT-DRY vs IMP INT-DRY	3.40	0.14	0.0009

Table S14. Pairwise compositional dissimilarities of biofilm bacterial communities within each stream. Comparisons were made between biofilms exposed to drying (CON-DRY, INT-DRY) and biofilms exposed sequentially to drying followed by WWTP effluent impacted (IMP CON-DRY, IMP INT-DRY).

Stream	Group	β_{SOR} (Sørensen)	β_{SIM} (Turnover)	β_{SNE} (Nestedness)
Arbúcies	CON DRY- IMP CON DRY	0.81	0.63	0.18
	INT DRY- IMP INT DRY	0.71	0.58	0.13
Llémena	CON DRY- IMP CON DRY	0.86	0.66	0.20
	INT DRY- IMP INT DRY	0.75	0.66	0.09
Major	CON DRY- IMP CON DRY	0.72	0.55	0.18
	INT DRY- IMP INT DRY	0.69	0.52	0.17
Terri	CON DRY- IMP CON DRY	0.59	0.53	0.05
	INT DRY- IMP INT DRY	0.48	0.44	0.04

Table S15. Results of the SIMPER analysis for block-wise treatment comparisons (unimpacted–drying, unimpacted–impacted, and drying–impacted) in each biofilm community. Bacterial families accounting cumulatively for up to 70% of the Bray–Curtis dissimilarity are listed. For each family, the mean SIMPER contribution, the percentage contribution, and the cumulative percentage are reported.

Treatment pairs	*Family*	*mean_contribution*	*perc_contrib*	*cumsum_perc*
**ARBÚCIES STREAM**				
**Unimpacted-Drying**	SepB-3	0.059	8.014	8.014
	Sphingomonadaceae	0.053	7.203	15.217
	Comamonadaceae	0.047	6.352	21.570
	Chloroplast	0.045	6.077	27.647
	Unknown_Family	0.043	5.814	33.461
	Rhizobiales_Incertae_Sedis	0.037	5.023	38.483
	Streptococcaceae	0.037	5.021	43.504
	Rhodobacteraceae	0.036	4.907	48.411
	Xenococcaceae	0.034	4.650	53.061
	Exiguobacteraceae	0.030	4.043	57.104
	Cyanobiaceae	0.021	2.912	60.016
	Deinococcaceae	0.018	2.411	62.427
	Leptolyngbyaceae	0.015	2.053	64.480
	Spirosomaceae	0.014	1.944	66.423
	Moraxellaceae	0.013	1.807	68.230
	Hyphomonadaceae	0.013	1.735	69.965
**Unimpacted-Impacted**	SepB-3	0.048	8.737	8.737
	Xenococcaceae	0.039	6.968	15.704
	Chloroplast	0.029	5.226	20.930
	Sphingomonadaceae	0.028	5.143	26.074
	Unknown_Family	0.028	5.045	31.119
	Rhodobacteraceae	0.026	4.741	35.860
	Streptococcaceae	0.026	4.662	40.522
	Comamonadaceae	0.025	4.568	45.090
	Exiguobacteraceae	0.021	3.748	48.838
	Moraxellaceae	0.019	3.464	52.302
	Rhizobiales_Incertae_Sedis	0.017	2.998	55.301
	Saprospiraceae	0.011	1.984	57.285
	Hyphomicrobiaceae	0.009	1.691	58.976
	Deinococcaceae	0.009	1.610	60.586
	Spirosomaceae	0.009	1.538	62.124
	Leptolyngbyaceae	0.008	1.514	63.638
	Hyphomonadaceae	0.008	1.376	65.015
	Cyanobiaceae	0.007	1.303	66.318
	Xanthomonadaceae	0.007	1.226	67.544
	Chromobacteriaceae	0.007	1.190	68.734
	Nitrosomonadaceae	0.006	1.132	69.866
**Drying-Impacted**	SepB-3	0.086	11.995	11.995
	Xenococcaceae	0.065	9.091	21.085
	Streptococcaceae	0.052	7.222	28.307
	Rhodobacteraceae	0.041	5.668	33.975
	Moraxellaceae	0.030	4.218	38.193
	Sphingomonadaceae	0.025	3.563	41.756

	Rhizobiales_Incertae_Sedis	0.024	3.414	45.170
	Comamonadaceae	0.019	2.713	47.883
	Saprospiraceae	0.019	2.628	50.511
	Hyphomicrobiaceae	0.016	2.251	52.762
	Chloroplast	0.016	2.228	54.990
	Cyanobiaceae	0.014	2.024	57.014
	Enterobacteriaceae	0.012	1.733	58.746
	Exiguobacteraceae	0.011	1.585	60.331
	Pseudomonadaceae	0.010	1.466	61.797
	Xanthomonadaceae	0.010	1.440	63.237
	Micrococcaceae	0.010	1.352	64.589
	Leuconostocaceae	0.009	1.245	65.833
	Xanthobacteraceae	0.009	1.192	67.025
	Nitrosomonadaceae	0.008	1.170	68.195
	Uncultured	0.008	1.105	69.300
**LLÉMENA STREAM**				
**Unimpacted-Drying**	Exiguobacteraceae	0.206	26.706	26.706
	Rhodobacteraceae	0.066	8.562	35.268
	Nostocaceae	0.056	7.245	42.513
	Sphingomonadaceae	0.038	4.921	47.433
	Micrococcaceae	0.035	4.573	52.007
	Oxalobacteraceae	0.029	3.714	55.721
	Intrasporangiaceae	0.023	2.937	58.657
	Rhizobiales_Incertae_Sedis	0.020	2.612	61.270
	Rubritaleaceae	0.019	2.423	63.693
	Pirellulaceae	0.016	2.121	65.813
	Chloroplast	0.016	2.115	67.928
	Steroidobacteraceae	0.014	1.840	69.768
**Unimpacted-Impacted**	Chloroplast	0.170	28.800	28.800
	Rhodobacteraceae	0.040	6.735	35.534
	Nostocaceae	0.036	6.116	41.651
	Sphingomonadaceae	0.022	3.795	45.446
	Mitochondria	0.020	3.317	48.763
	Hyphomicrobiaceae	0.017	2.933	51.696
	Gemmataceae	0.017	2.851	54.547
	Chamaesiphonaceae	0.017	2.843	57.390
	Phormidiaceae	0.015	2.534	59.924
	Pirellulaceae	0.014	2.405	62.330
	Rhizobiales_Incertae_Sedis	0.012	2.084	64.414
	Isosphaeraceae	0.012	2.016	66.430
	Micrococcaceae	0.010	1.622	68.052
	Exiguobacteraceae	0.009	1.580	69.632
**Drying-Impacted**	Chloroplast	0.181	22.498	22.498
	Exiguobacteraceae	0.139	17.289	39.787
	Rhodobacteraceae	0.056	7.003	46.790
	Sphingomonadaceae	0.027	3.299	50.089
	Hyphomicrobiaceae	0.022	2.748	52.837
	Gemmataceae	0.022	2.746	55.584
	Pirellulaceae	0.021	2.587	58.171

	Mitochondria	0.021	2.547	60.718
	Rhizobiales_Incertae_Sedis	0.020	2.446	63.165
	Oxalobacteraceae	0.019	2.349	65.514
	Micrococcaceae	0.017	2.134	67.648
	Chamaesiphonaceae	0.017	2.099	69.747

MAJOR STREAM

**Unimpacted-Drying**	Unknown_Family	0.169	21.332	21.332
	Comamonadaceae	0.064	8.035	29.367
	Chloroplast	0.059	7.413	36.781
	Exiguobacteraceae	0.046	5.804	42.584
	Micrococcaceae	0.041	5.206	47.790
	Oxalobacteraceae	0.038	4.774	52.564
	Rhodobacteraceae	0.036	4.507	57.071
	Chamaesiphonaceae	0.031	3.975	61.046
	Rhizobiales_Incertae_Sedis	0.025	3.135	64.181
	Sphingomonadaceae	0.022	2.818	66.999
	Sphingobacteriaceae	0.021	2.641	69.640
**Unimpacted-Impacted**	Unknown_Family	0.120	21.681	21.681
	Comamonadaceae	0.067	12.102	33.784
	Pseudomonadaceae	0.045	8.211	41.995
	Chloroplast	0.034	6.150	48.145
	Methylophilaceae	0.031	5.592	53.737
	Moraxellaceae	0.028	5.023	58.760
	Rhodobacteraceae	0.024	4.352	63.112
	Nocardiaceae	0.019	3.424	66.535
	Sphingomonadaceae	0.016	2.907	69.443
**Drying-Impacted**	Comamonadaceae	0.119	16.840	16.840
	Rhodobacteraceae	0.056	7.949	24.789
	Pseudomonadaceae	0.053	7.461	32.250
	Exiguobacteraceae	0.043	6.052	38.302
	Methylophilaceae	0.041	5.848	44.151
	Moraxellaceae	0.037	5.197	49.347
	Oxalobacteraceae	0.034	4.747	54.094
	Micrococcaceae	0.031	4.435	58.529
	Nocardiaceae	0.023	3.270	61.799
	Sphingobacteriaceae	0.020	2.834	64.633
	Chamaesiphonaceae	0.019	2.695	67.328
	Sphingomonadaceae	0.017	2.338	69.666

TERRI STREAM

**Unimpacted-Drying**	Exiguobacteraceae	0.077	17.402	17.402
	Xenococcaceae	0.046	10.549	27.951
	Chloroplast	0.032	7.210	35.162
	Intrasporangiaceae	0.029	6.629	41.791
	Comamonadaceae	0.023	5.329	47.119
	Rhodobacteraceae	0.019	4.329	51.448
	Sphingomonadaceae	0.015	3.458	54.906

	Oxalobacteraceae	0.011	2.496	57.402
	Caulobacteraceae	0.010	2.238	59.640
	SepB-3	0.009	2.028	61.668
	Rhizobiales_Incertae_Sedis	0.008	1.839	63.507
	Nocardioidaceae	0.007	1.677	65.184
	Chamaesiphonaceae	0.006	1.321	66.504
	Xanthomonadaceae	0.006	1.286	67.790
	Spirosomaceae	0.005	1.237	69.028
**Unimpacted-Impacted**	Xenococcaceae	0.039	11.540	11.540
	Chloroplast	0.029	8.586	20.126
	SepB-3	0.027	7.971	28.098
	Rhodobacteraceae	0.019	5.584	33.682
	Sphingomonadaceae	0.017	5.128	38.810
	Comamonadaceae	0.015	4.526	43.335
	Methylophilaceae	0.010	3.098	46.433
	Nocardiaceae	0.010	3.060	49.493
	Xanthomonadaceae	0.009	2.526	52.019
	Hyphomicrobiaceae	0.008	2.336	54.355
	Rhizobiales_Incertae_Sedis	0.006	1.683	56.038
	Flavobacteriaceae	0.005	1.539	57.577
	Chamaesiphonaceae	0.005	1.491	59.068
	Alcanivoracaceae	0.005	1.459	60.527
	Phormidesmiaceae	0.005	1.429	61.956
	Mycobacteriaceae	0.005	1.389	63.345
	Blastocatellaceae	0.005	1.377	64.722
	Leptolyngbyaceae	0.004	1.314	66.036
	Moraxellaceae	0.004	1.297	67.333
	Exiguobacteraceae	0.004	1.200	68.533
	Sutterellaceae	0.004	1.150	69.682
**Drying-Impacted**	Exiguobacteraceae	0.067	16.561	16.561
	Xenococcaceae	0.033	8.015	24.576
	SepB-3	0.028	6.821	31.396
	Intrasporangiaceae	0.025	6.144	37.541
	Comamonadaceae	0.022	5.506	43.047
	Sphingomonadaceae	0.016	4.014	47.061
	Rhodobacteraceae	0.014	3.522	50.584
	Methylophilaceae	0.010	2.515	53.098
	Oxalobacteraceae	0.010	2.437	55.535
	Hyphomicrobiaceae	0.009	2.235	57.770
	Nocardiaceae	0.008	2.094	59.864
	Caulobacteraceae	0.008	1.920	61.785
	Rhizobiales_Incertae_Sedis	0.007	1.847	63.632
	Nocardioidaceae	0.006	1.495	65.127
	Xanthomonadaceae	0.005	1.344	66.471
	Alcanivoracaceae	0.005	1.288	67.759
	Chitinophagaceae	0.005	1.284	69.043
